# Learning Effective Connectivity Network Structure from fMRI Data Based on Artificial Immune Algorithm

**DOI:** 10.1371/journal.pone.0152600

**Published:** 2016-04-05

**Authors:** Junzhong Ji, Jinduo Liu, Peipeng Liang, Aidong Zhang

**Affiliations:** 1 Beijing Municipal Key Laboratory of Multimedia and Intelligent Software, College of Computer Science and Technology, Beijing University of Technology, Beijing, China; 2 Beijing Key Lab of MRI and Brain Informatics, Department of Radiology, Xuanwu Hospital, Capital Medical University, Beijing, China; 3 Department of Computer Science and Engineering, University at Buffalo, The State University of New York, Buffalo, United States of America; Duke-NUS Graduate Medical School, SINGAPORE

## Abstract

Many approaches have been designed to extract brain effective connectivity from functional magnetic resonance imaging (fMRI) data. However, few of them can effectively identify the connectivity network structure due to different defects. In this paper, a new algorithm is developed to infer the effective connectivity between different brain regions by combining artificial immune algorithm (AIA) with the Bayes net method, named as AIAEC. In the proposed algorithm, a brain effective connectivity network is mapped onto an antibody, and four immune operators are employed to perform the optimization process of antibodies, including clonal selection operator, crossover operator, mutation operator and suppression operator, and finally gets an antibody with the highest K2 score as the solution. AIAEC is then tested on Smith’s simulated datasets, and the effect of the different factors on AIAEC is evaluated, including the node number, session length, as well as the other potential confounding factors of the blood oxygen level dependent (BOLD) signal. It was revealed that, as contrast to other existing methods, AIAEC got the best performance on the majority of the datasets. It was also found that AIAEC could attain a relative better solution under the influence of many factors, although AIAEC was differently affected by the aforementioned factors. AIAEC is thus demonstrated to be an effective method for detecting the brain effective connectivity.

## Introduction

Effective connectivity is the influence that one neuronal system exerts over another between brain regions [1]. Effective connectivity is different from functional connectivity, and can render the performance of the specific tasks under conditions of functional connectivity. Specifically, effective connectivity can describe the directed networks in the resting state and specific changes of baseline brain activity in some diseases [2, 3]. How to accurately identify effective connectivity from functional magnetic resonance imaging (fMRI) data is becoming a research hotspot in the domain of neuroimaging as well as cognitive neuroscience.

Recently, various mathematical methods have been widely used to determine the effective connectivity involved in human brain [4]. One kind of these methods is the model-driven approach or hypothesis-driven approach, such as structural equation modeling (SEM) [5] and dynamic causal modeling (DCM) [6]. The priori models are required for this method to conduct a valid connectivity analysis. The model-driven approach is thus not suitable for resting-state fMRI data or for those situations where the prior knowledge is insufficient [7–9]. In particular, the model-driven approach is typically limited to construct the relative small networks, and does not have the ability to effectively search across the full range of possible network topologies.

Another kind of effective connectivity methods are the data-driven approaches. The data-driven approaches directly extract causal interactions from fMRI data, but do not require the prior knowledge or assumptions. However, different types of data-driven methods still have their own limitations. For example, Granger causality uses a vector autoregressive model to estimate the effective connectivity among brain regions [10, 11], and only requires the data to be wide-sense stationary and has a zero mean [12]. However, Granger causality is sensitive to noise and down sampling, thus it may generate spurious causality under some circumstances [13]. Linear non-Gaussian acyclic model (LiNGAM) [14] algorithm utilizes higher-order distributional statistics and independent component analysis (ICA) to estimate the network connections. Nevertheless, some prior assumptions are required by LiNGAM [15], including: (a) the data generating process is linear, (b) no unobserved confounders are present, and (c) disturbance variables follow non-Gaussian distributions. These assumptions per se have limited its use [8]. Generalised synchronization (Gen Synch) [16] evaluates neural synchrony by analyzing the interdependence between the signals, and employs three related measures of nonlinear interdependence, called *S*^*k*^, *H*^*k*^, *N*^*k*^ [17]. The three measures generated by Gen Synch are directional, but the direction of the asymmetry is not always consistent [8]. Patel’s conditional dependence measures use a multinomial likelihood with a Dirichlet prior distribution to construct a bivariate Bernoulli Bayesian model for the joint activation of each pair of brain voxels, and formulates a measure of connection strength *κ* and a measure of connection directionality *τ* [18]. Although Patel’s *τ* is demonstrated to be prior to the other methods at identifying the directions which can reach nearly 65% at d-accuracy [8], it should be further improved, as Patel’s *κ* performs worse than the partial correlation, inverse covariance (ICOV), as well as Bayes net methods at c-sensitivity.

Bayes net is another kind of data-driven approaches for identifying the effective connectivity [19–21]. Many Bayes net methods have been developed, such as PC [22], conservative PC (CPC) [23], cyclic causal discovery (CCD) [24], fast causal inference (FCI) [25], greedy equivalence search (GES) [26] and independent multisample greedy equivalence search (iMaGES) [27]. It was found that Bayes net methods, e.g. PC and GES, performed well in identifying functional connectivity, but none of them completely and reliably inferred causal directions [8]. One possible reason may be ascribed to the fact that these Bayes net methods have less search ability in the space of the candidate network topologies. So far, how to further explore new Bayes net modeling methods for identifying effective connectivity from fMRI data is still a challenging research topic.

In this paper, a new method for learning effective connectivity network structure from fMRI data is presented by combining artificial immune algorithm (AIA) with the Bayes net method, named as AIAEC. The focus of the algorithm is the optimization process of antibody population where some artificial immune mechanisms are employed to iteratively search for the best effective connectivity network structure. During each iteration, AIAEC first makes up an initial population including memorized antibodies and randomly generated antibodies, and computes an affinity value for every antibody. Then three operators of clonal selection, crossover, and mutation are performed to optimize antibodies in the current population. Finally, AIAEC updates antibodies in the current population by a suppression operator, and obtains new memorized antibodies. This iteration process is repeated until the best solution is found. A series of experiments have been carried out on all Smith’s simulated datasets of 50 subjects.

## Methods

### Artificial Immune Algorithm

The human immune system is a remarkable information processing and self learning system in nature. Inspired by the human immune system, an artificial immune system (AIS) is built to solve some complex computational problems [28, 29]. In the last decade, AIS has drawn significant attention and obtained widespread development and application. Especially, its highly distributed, adaptive, and self-organizing nature, together with its learning, memory, feature extraction, and pattern recognition, always offers rich metaphors for novel approaches to many real-world problems [30].

As a main form of AIS, AIA receives inspiration from the cell theory and network theory, and implements antigen recognition, cell differentiation, memory and the self adjustment functions in the immune system. In general, AIA roughly contains the following steps: 1) Randomly generate an initial population, 2) Calculate the affinity of the antibodies in a population, 3) Select some antibodies with higher affinity values and then clone them, 4) Mutate these antibodies which are generated by clone, and 5) Update the population. This process is repeated until a termination criterion is satisfied. A general artificial immune algorithm is shown in Algorithm 1.

**Algorithm 1** Artificial Immune Algorithm

**Begin**

 Initialize population;

**Repeat**

 Evaluate the population: calculate the affinity of every antibody to antigen;

 Perform immune operations;

 { 1) Clone operation: Select some antibodies and then clone them;

  2) Mutate operation: Mutate the generated clones; }

 Update the population;

**Until** requirements are met

**End**

In this paper, based on AIA, we present a new algorithm, named as AIAEC to learn an effective connectivity network structure from fMRI data based on K2 scoring metric (see below in Formula (1) for definition). The description of AIAEC is as follows.

### The AIAEC algorithm

**Fig 1:**
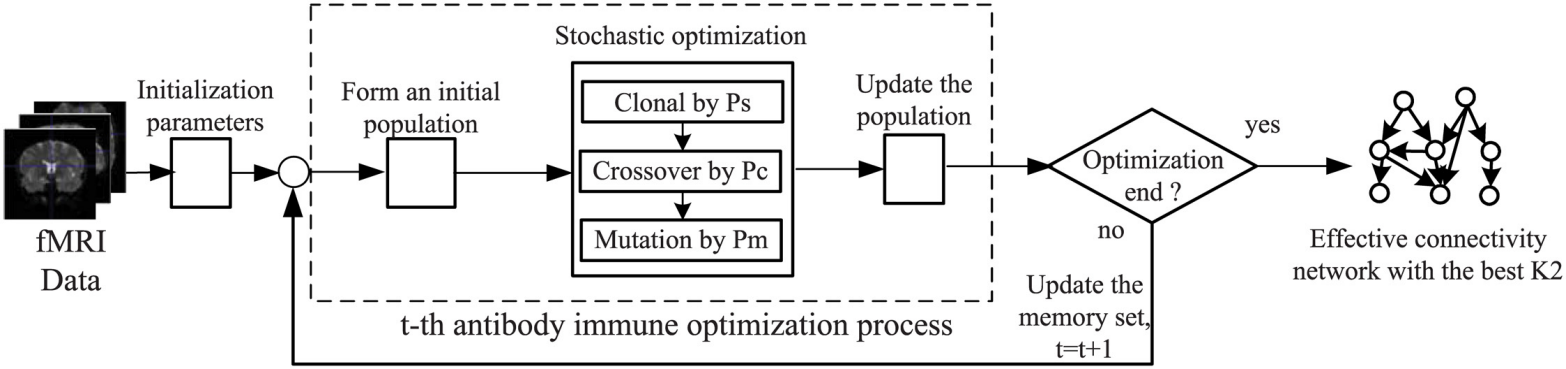
The flowchart of the proposed AIAEC algorithm, where an effective connectivity network with the best K2 is obtained by the antibody immune optimization process.

In this section, we give a detailed description of the AIAEC algorithm, and introduce how to learn an effective connectivity from fMRI data. AIAEC algorithm is a score-and-search approach, which is based on an artificial immune principle for determining the structure of brain effective connectivity network. Just like many methods based on Bayes net, this paper also views an effective connectivity network as a directed acyclic graph (DAG). AIAEC is essentially a global search method to learn Bayesian network structure, where every solution represents an effective connectivity network. Fig 1 shows the flowchart of the proposed algorithm. In AIAEC, we first map a brain effective connectivity network into an antibody in the artificial immune system, and employ the K2 metric (see below in Formula (1) for definition) used in Bayesian network learning to evaluate the affinity of every antibody in a population and guide the optimization process to search for the global maximum in a feasible solution space. To simulate the artificial immune mechanism, we develop some immune operators to get some antibodies with the higher score in each iteration. Once end requirements are met, the antibody with the highest score in the optimization process is reversely mapped to the real brain effective connectivity network.

#### Representation of the problem

**Fig 2:**
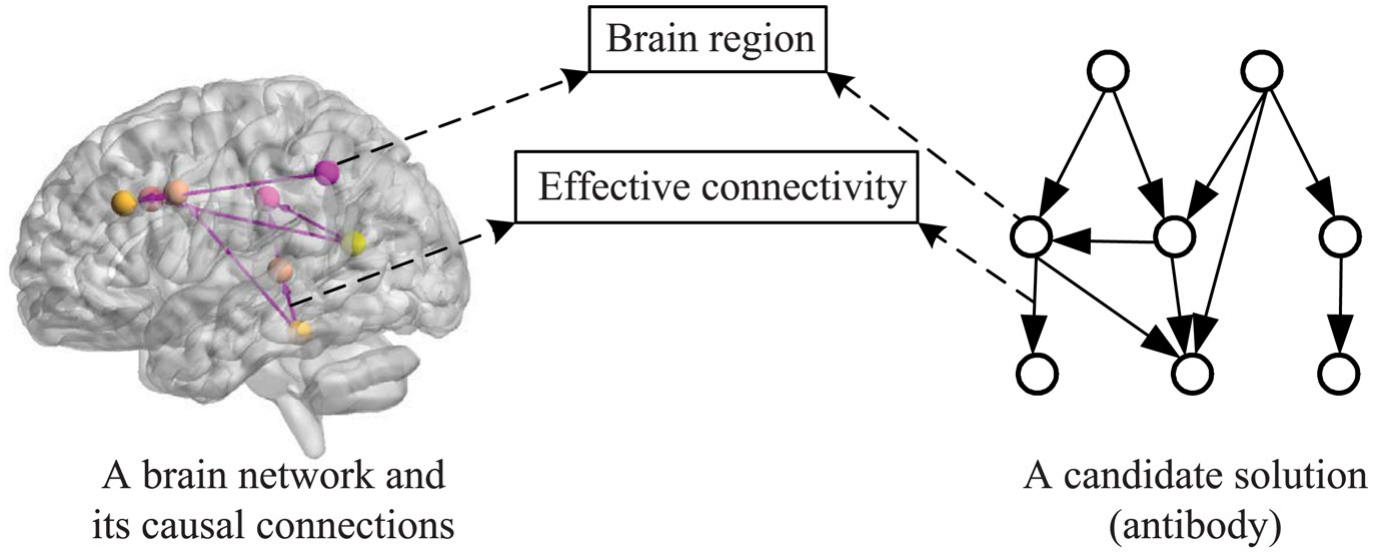
The mapping relationship between a brain network and its corresponding candidate solution.

Identifying effective connectivity network structure by AIAEC in essence is a discrete optimization problem. Fig 2 gives the mapping relationship between a brain network and its corresponding candidate solution, where the representation of the problem is a graph, the states (solutions) of the problem are DAGs with a set of *n* nodes (**X**), each node *X*_*i*_ ∈ **X** denotes a brain region, and each arc *a*_*ij*_ shows a causal connection between two brain regions *X*_*i*_ and *X*_*j*_. Thus, a solution *G*_*k*_ will be a graph including a set of nodes (**X**), a set of arcs (**A**) and no directed cycle. In AIAEC, every antibody in a population represents such a candidate solution.

#### Solution construction

**Fig 3:**
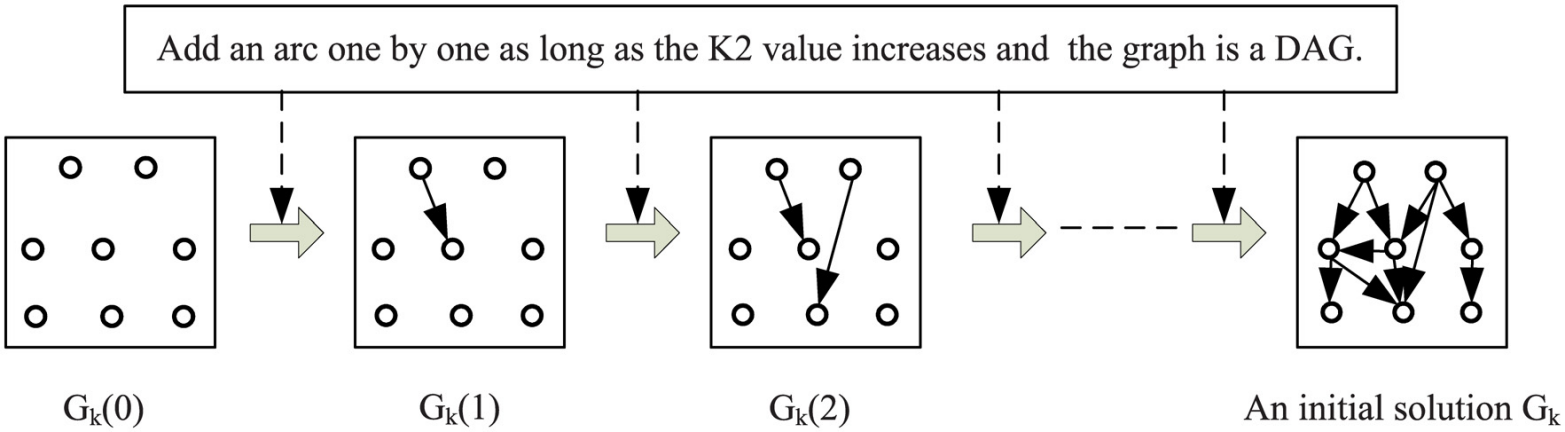
The schematic diagram of the process of constructing a solution, where an arc is added one by one from an empty graph to an initial solution (DAG).

In each iteration, antibodies in the initial population are composed of *M* antibodies in a memory set and *N* − *M* new antibodies, where the memory set stores the best *M* antibodies obtained so far, *N* is the population size of antibodies, and each new antibody is randomly generated by a solution construction process. The construction process is showed in Fig 3, where starting from an empty graph with no edge, an arc absent in the current graph is added to the solution one by one if and only if the *K*2 score of the new solution is larger than that of the old one and the generated graph satisfies the DAG constraint. This process is repeated until there is no way to make the *K*2 score of the new solution higher by adding an arc. In the first iteration, since the memory set is empty, all *N* antibodies in the initial population are randomly generated entirely.

#### Affinity metric of an antibody

To evaluate whether antibodies are well matched for antigens, an affinity metric is employed to evaluate the quality of the generated antibodies. In AIAEC, we employ an antibody to represent a DAG, and use the *K*2 metric to evaluate the affinity of an antibody. The K2 metric is well-known as a structure score in Bayesian network learning, which can present the interesting characteristic by expressing a tradeoff between quality and complexity, and favor networks with higher likelihood and simpler structures [31]. The expression of the *K*2 metric is:

$$\begin{array}{c} P\left(G,Data\right)=P\left(G\right)\cdot \prod_{i=1}^{n}\prod_{j=1}^{q_{i}}\frac{(r_{i}-1)!}{(N_{ij}+r_{i}-1)!}\prod_{k=1}^{r_{i}}N_{ijk}!, \end{array}$$ (1)

where *G* is a possible network structure, *Data* is the fMRI data set discretized, *r*_*i*_ is the number of possible values of the node variable *X*_*i*_, *q*_*i*_ is the number of possible configurations (instantiations) for the node variables in ∏(*X*_*i*_), and *N*_*ijk*_ is the number of cases in *Data* with *X*_*i*_ has its *k*^*th*^ value and ∏(*X*_*i*_) is instantiated to its *j*^*th*^ configuration. From the perspective of the meaning of the formula, the best *K*2 value is the biggest one which is related to the optimal structure of an effective connectivity network on *Data*.

#### Immune operator

**Fig 4:**
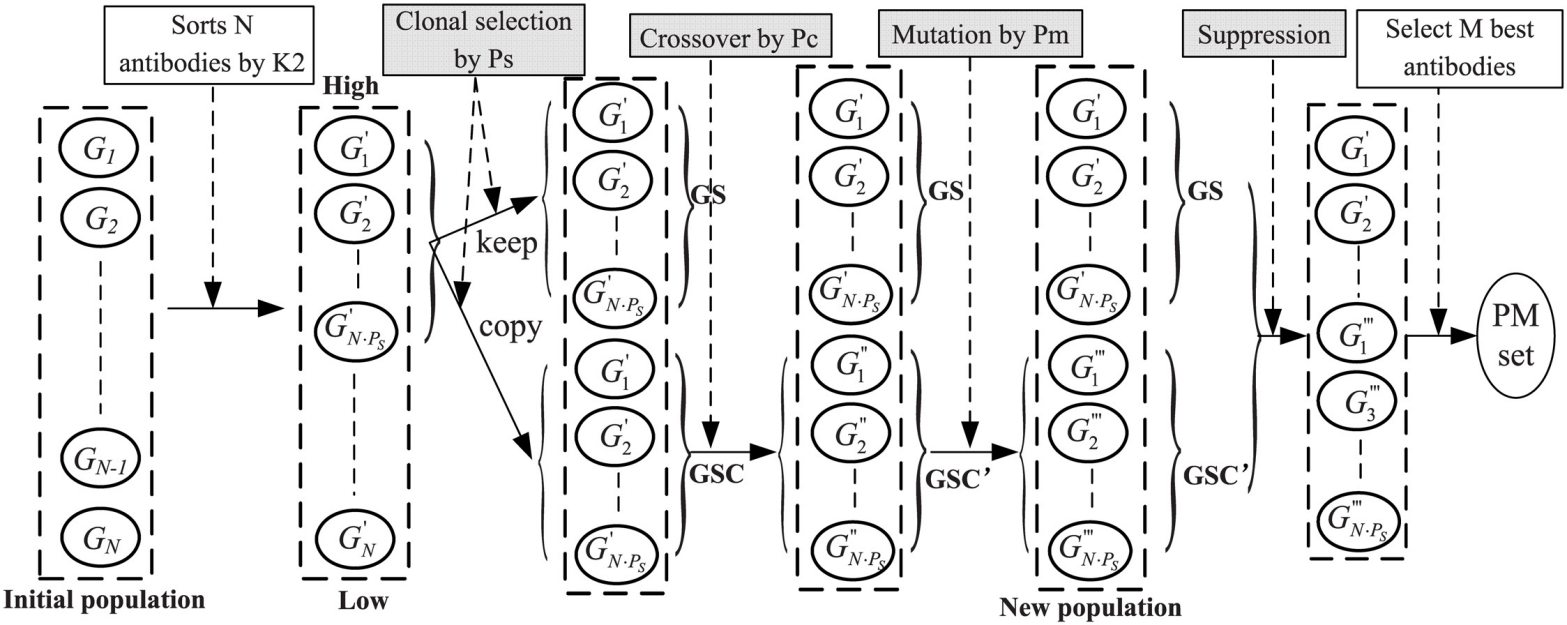
The schematic diagram of the optimization process of antibodies, where four immune operators are employed to optimize antibodies.

After the initial population is formed in each iteration, antibodies in the population will randomly perform some immune operators to search better antibodies (solutions). To perform the optimization process of antibodies in AIAEC, we employ four immune operators, namely clonal selection operator, crossover operator, mutation operator, and suppressing operator. Fig 4 shows the schematic diagram of the optimization process of antibodies in a population, where these shaded areas represent the four immune operators. In the following, we will give the detailed descriptions about them.

**1) Clonal selection operator**. The excellent antibodies always have a good ability to adapt to the environment, so the number of excellent antibodies will increase along with the evolution of antibodies. The clonal selection operator is to select some antibodies with higher affinity values from the initial population, and keep them and their derivatives generated by crossover and mutation operators into the updating population at the current iteration.

As shown in Fig 4, the operator first sorts the antibodies in the initial population by their affinity values (K2 values), and selects *N* ⋅ *P*_*s*_ antibodies with the biggest affinity values as a set of selected antibodies (**GS**), where *P*_*s*_ is a probability of clonal selections. Then all selected antibodies are completely cloned to form a set of copied antibodies (**GSC**). Obviously, **GS** = **GSC** when the clonal selection operator is just finished. Then, the crossover and mutation operators are executed on some antibodies in **GSC** to search for better antibodies. In a word, clonal selection operator retains some excellent antibodies, and provides the possibility for these antibodies to change better in each iteration.

**Fig 5:**
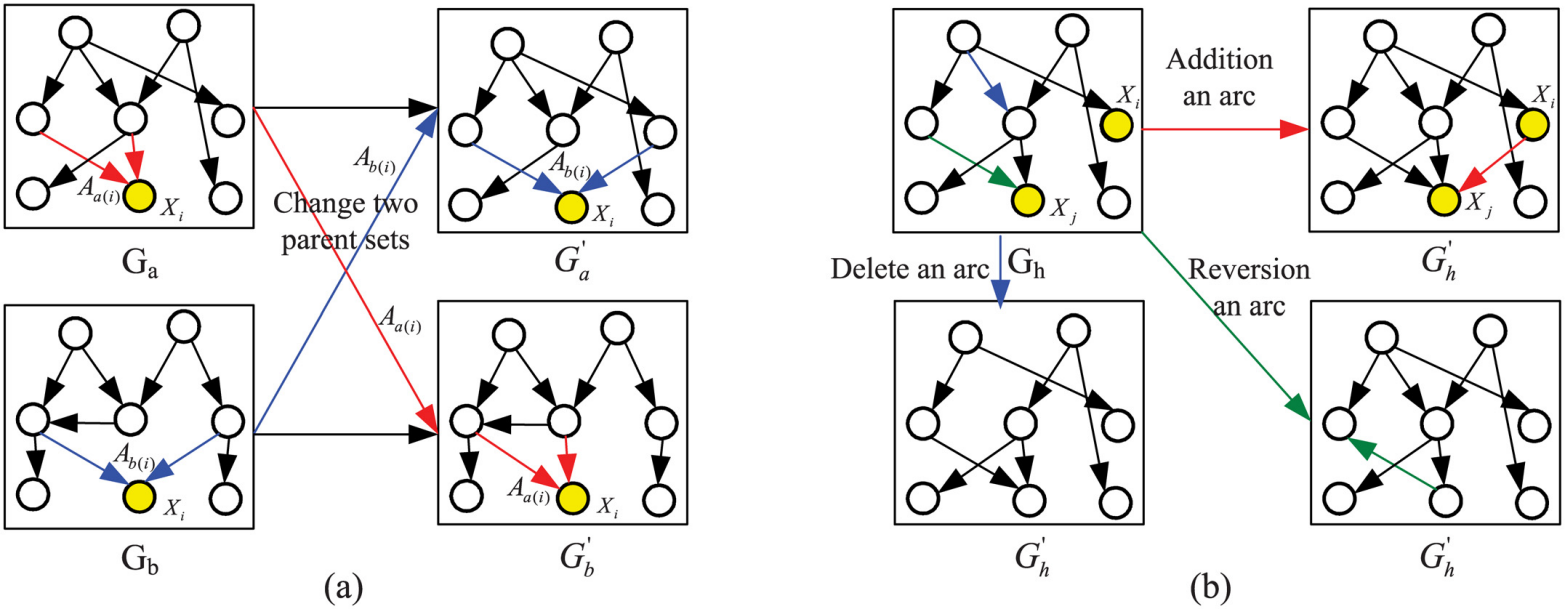
The sample graphs of the crossover and mutation operators. (a) Crossover operator. (b) Mutation operator.

**2) Crossover operator**. Crossover refers to that two parent antibodies generate two new antibodies by locally exchanging antibody components between the two parent antibodies. As shown in Fig 5(a), suppose that two parent antibodies are *G*_*a*_ and *G*_*b*_ in **GSC**, *X*_*i*_ is a shared node in *G*_*a*_ and *G*_*b*_, *A*_*a*(*i*)_ and *A*_*b*(*i*)_ are two arc sets connected to *X*_*i*_ in *G*_*a*_ and *G*_*b*_, respectively, and *A*_*a*(*i*)_ ≠ *A*_*b*(*i*)_. To obtain offsprings of the two parent antibodies, the rule of the crossover operator is designed as follows: If exchanging *A*_*a*(*i*)_ and *A*_*b*(*i*)_ between *G*_*a*_ and *G*_*b*_ still forms two directed acyclic graphs, i.e., $G_{a}^{\prime }$ and $G_{b}^{\prime }$, then $G_{a}^{\prime }=G_{a}\backslash A_{a\left(i\right)}\cup A_{b\left(i\right)}$, $G_{b}^{\prime }=G_{b}\backslash A_{b\left(i\right)}\cup A_{a\left(i\right)}$, and *G*_*a*_ and *G*_*b*_ in **GSC** are replaced with $G_{a}^{\prime }$ and $G_{b}^{\prime }$. It should be noted that two parent antibodies and their shared node are randomly selected from **GSC** and the set of nodes, respectively, which ensures the randomness and diversity of new antibodies. Based on a crossover probability *P*_*c*_, this crossover operator is repeated *N* ⋅ *P*_*s*_ ⋅ *P*_*c*_ times in each iteration. Obviously, the crossover operator has the function of a random search, which is performed on parent antibodies to achieve the purpose of cooperation with a crossover probability *P*_*c*_.

**3) Mutation operator**. Mutation is a structure change of an antibody in its neighbor solution space. For a solution *G*_*h*_ in **GSC**, AIAEC employs addition, deletion, and reversion strategies to carry out the mutation operator, where the constraint of directed acyclic is always remained. All these strategies on the current solution will generate a new solution by simply modifying the set of arcs **A** in *G*_*h*_. Fig 5(b) gives three instances of these mutation strategies, which can be described as:

- Addition: The strategy randomly selects two nodes *X*_*j*_ and *X*_*i*_ in *G*_*h*_ where *i* ≠ *j*, and *X*_*i*_ ∈ **X** \ *Π*(*X*_*j*_). If adding an arc *a*_*ij*_ = *X*_*i*_ → *X*_*j*_ does not generate a directed cycle, then $G_{h}^{\prime }=G_{h}\cup \left\{a_{ij}\right\}$.
- Deletion: The strategy first randomly selects an arc *a*_*ij*_ ∈ **A** which is present in *G*_*h*_, then deletes it from the *G*_*h*_. Namely, a new solution, $G_{h}^{\prime }=G_{h}\setminus \left\{a_{ij}\right\}$, is obtained.
- Reversion: The strategy randomly selects an arc *a*_*ij*_ ∈ **A**, and then modifies the direction of the arc if the reversion of the arc in *G*_*h*_ still forms a DAG. By means of this strategy, a new solution, $G_{h}^{\prime }=G_{h}\backslash \left\{a_{ij}\right\}\cup \left\{a_{ji}\right\}$, is obtained.

Based on a mutation probability *P*_*m*_, the mutation operator is performed *N* ⋅ *P*_*s*_ ⋅ *P*_*m*_ times in each iteration. Each mutation randomly selects one of three strategies to carry out while keeping the constraint of any directed acyclic graph. Once a mutation operator is performed, the current solution in **GSC** will be replaced with the solution newly generated. By mutation operator, an antibody can implement self-changing to get better in each iteration.

**4) Suppression operator**. Updating the population is an important step to search for good solutions in every iteration. The new population at every iteration consists of two parts: all antibodies in **GS** and all antibodies in **GSC**. Though many antibodies in **GSC** have been changed by the crossover and mutation operators, there might be some antibodies in **GSC** which are the same as antibodies in **GS**. To avoid redundancy and maintain the diversity of antibodies, suppression operator is employed to eliminate identical antibodies in the new population. Since each antibody structure will get an affinity value (*K*2 score), the suppression method is designed to compute the affinity value for each changed antibody in **GSC** and then compare affinity values of all antibodies in the new population. For those antibodies with the same affinity value, we only retain one of them and remove the others from the new population. So suppression operator is employed to eliminate the duplicate antibodies to maintain the diversity of population.

### AIAEC algorithm

The proposed AIAEC algorithm is presented in Algorithm 2. It starts with an initialization phase where some parameters are preset. Then an antibody optimization process is performed where four artificial immune mechanisms are employed to search an optimal solution. In each iteration, there are 8 steps as follows: 1) An initial population P[t] is generated, it not only includes high-quality solutions in the past memory, but also adds new solutions randomly generated; 2) Every antibody in the population is evaluated using the K2 metric to test its own affinity; 3) Clonal selection operator first selects a set **GS** to keep some high-quality solutions, then completely clones these antibodies in **GS** and forms a copy set **GSC** to perform further immune operators; 4) Based on *P*_*c*_, crossover operators on antibodies in **GSC** are carried out, and original antibodies involved in crossover operators are replaced with antibodies generated; 5) Based on *P*_*m*_, mutation operators on antibodies in **GSC** are executed, and similarly original antibodies involved in mutation operators are also replaced with antibodies generated; 6) The initial population is updated with antibodies in **GS** and **GSC**; 7) Suppression operator is employed to remove redundancy antibodies in the current population; and 8) Memory mechanism selects the best *M* antibodies in the current P[t] to update the memory set PM[t]. This process is repeated until the termination criterion is satisfied. In AIAEC, the algorithm terminates when the iteration of the antibodies achieves the maximum number of iterations (T). Finally, AIAEC returns the solution with the highest K2 value in all iterations as the output result.

**Algorithm 2** AIAEC: Artificial Immune Algorithm to identify Effective Connectivity

**Input**: fMRI Data

**Output**: Brain effective connectivity network

**1**. **Initialization**:

 Set parameters N, T, M, *P*_*s*_, *P*_*c*_, *P*_*m*_, PM[0] = *ϕ*;

  *N: population size of antibodies, T: maximum number of iterations, *

  *M: capacity of the memory set, *P*_*s*_: probability of clonal selections, *

  *P_*c*_: probability of crossovers, *P*_*m*_: probability of mutations, *

  *PM[0]: initial memory set with best antibodies. *

**2**. **Loop: Antibody optimization process**

 For t = 0 to T *t is the iteration number of antibodies*

  { 1) Generate an initial population P[t]

   P[t] = PM[t] + PG[t]; * PG[t]: set of antibodies randomly generated *

  2) Calculate the affinity value for every antibody

   For k = 0 to N

    Compute the K2 value of *G*_*k*_ ∈ *P*[*t*] by Equ.1;

  3) Perform clonal selection operator

   Select and obtain a set of *N* ⋅ *P*_*s*_ antibodies with the higher K2 values (**GS**) by *P*_*s*_;

   Clone these selected antibodies, and form a copy set **GSC**;

  4) Perform a crossover operator

   For i = 1 to |**GSC**| ⋅ *P*_*c*_

    { Select two antibodies from **GSC**, and perform crossover operator;

    Update **GSC** with new antibodies generated; }

  5) Perform a mutation operator

   For i = 1 to |**GSC**| ⋅ *P*_*m*_

    { Select an antibody from **GSC**, and perform a mutation operator;

    Update **GSC** with the antibody newly generated; }

  6) Update the population

   P[t] = **GS**+**GSC**;

  7) Perform suppression operator

   P[t] = P[t]-{*G*_*j*_|∀*G*_*i*_ ∈ *P*[*t*], *i* ≠ *j*, *and G*_*i*_ = *G*_*j*_};

  8) Memorize the solutions with higher affinity values

   Put the best M antibodies in *P*[*t*] into the memory set PM[t + 1];

   t = t + 1; }

**3**. **Return**: Effective connectivity network with the highest *K*2 value;

In essence, AIAEC algorithm uses crossover and mutation operators to locally optimize solutions in the current population, and employs an exploring phase of randomly generated solutions to overcome the stagnation of solutions in the whole optimization, which not only keeps the balance between exploitation and exploration processes, but also realizes the perfect combination of global searching and local searching in the available solution space. Moreover, clonal and memory mechanisms play an important role in the transfer of good solution information. Specifically, clonal mechanism selects some good antibodies as starting points to be further searched while memory mechanism reserves the best antibodies into the next iteration.

## Results

In this study, Smith et al. (2011) simulated datasets (http://www.fmrib.ox.ac.uk/analysis/netsim/index.html) are used to test the proposed AIAEC algorithm. The experimental platform is a PC with Core 2, 2.13 GHz CPU, 2.99 GB RAM, and Windows 7. The performances of AIAEC on the simulated datasets are assessed, and then compared with the other 10 existing algorithms. Seven of them including PC, CPC, CCD, FCI, GES, iMaGES, and LiNGAM are implemented in the Tetrad IV toolbox (www.phil.cmu.edu/projects/tetrad/tetrad4.html). Granger and Gen Synch are run from two corresponding public platforms (www.mathworks.co.kr/matlabcentral/fileexchange/25467-grangercausality-test and www.vis.caltech.edu/rodri/programs/synchro.m), respectively. Additionally, Patel is directly accomplished from Smith [8].

### Simulation of fMRI Data

The data derived from 28 simulation cases was created with different number of nodes and percent of noise [8]. The nodes are corresponded to brain regions, and the simulated networks contain 5, 10, 15 or 50 nodes, respectively. The blood oxygen level dependent (BOLD) data was sampled with a repetition time (TR) of 3 s (reduced to 0.25 s in a few simulations), and all the simulations comprised 50 separate subjects where most of them employed the same simulation parameters. Moreover, each subject’s data was a 10-min fMRI session (200 time points) in most of the simulations. In this experiment, the BOLD time series data are concatenated over 50 subjects and analyzed for each simulated dataset. Table 1 shows a summary of the specifications for the 28 simulated datasets.

**Table 1:** Description of the 28 simulation cases [8].

Sim	nodes	Session duration (min)	TR (s)	Noise (%)	HRF std. dev. (s)	Other factors
1	5	10	3.00	1.0	0.5	
2	10	10	3.00	1.0	0.5	
3	15	10	3.00	1.0	0.5	
4	50	10	3.00	1.0	0.5	
5	5	60	3.00	1.0	0.5	
6	10	60	3.00	1.0	0.5	
7	5	250	3.00	1.0	0.5	
8	5	10	3.00	1.0	0.5	shared inputs
9	5	250	3.00	1.0	0.5	shared inputs
10	5	10	3.00	1.0	0.5	global mean confound
11	10	10	3.00	1.0	0.5	bad ROIs (timeseries mixed with each other)
12	10	10	3.00	1.0	0.5	bad ROIs (new random timeseries mixed in)
13	5	10	3.00	1.0	0.5	backwards connections
14	5	10	3.00	1.0	0.5	cyclic connections
15	5	10	3.00	0.1	0.5	stronger connections
16	5	10	3.00	1.0	0.5	more connections
17	10	10	3.00	0.1	0.5	
18	5	10	3.00	1.0	0.0	
19	5	10	0.25	0.1	0.5	neural lag = 100 ms
20	5	10	0.25	0.1	0.0	neural lag = 100 ms
21	5	10	3.00	1.0	0.5	2-group test
22	5	10	3.00	0.1	0.5	nonstationary connection strengths
23	5	10	3.00	0.1	0.5	stationary connection strengths
24	5	10	3.00	0.1	0.5	only one strong external input
25	5	5	3.00	1.0	0.5	
26	5	2.5	3.00	1.0	0.5	
27	5	2.5	3.00	0.1	0.5	
28	5	5	3.00	0.1	0.5	

### Preprocessing

Like many other Bayesian network learning algorithms, a discrete processing is essential for AIAEC, as it cannot directly use continuous variables. According to the number of time points, the discretized instance data are obtained for the whole brain, where each instance includes the discretized values of all brain regions (nodes) at the corresponding time point. For each node’s timeseries of a subject, the range of voxel values is divided into several equal parts, and each part contains the same number of voxel values. Based on the division of node values, the voxel value of each node is quantized at every instance into a discrete value. For example, a node’s time series is quantized into four parts, including low (set value = 1), medium (set value = 2), high (set value = 3) and very high (set value = 4), with each of the four parts containing 25% of the data points. In this experiment, the number of discrete parts for the 28 simulated datasets is varied from 3 to 8.

### Evaluation metrics

In Smith et al. (2011), they use “c-sensitivity” and “d-accuracy” to evaluate the network connection and the connection direction. To more clearly evaluate the performance of algorithms, we use Precision, Recall and F-measure to measure the network connection and direction. Connection’s Precision and Recall can be defined as follows:

$$\begin{array}{c} Precision_{c}=\frac{C_{s}}{C_{a}+C_{s}} \end{array}$$ (2)

and

$$\begin{array}{c} Recall_{c}=\frac{C_{s}}{TC}, \end{array}$$ (3)

where *C*_*a*_, *C*_*s*_ are used to show the structure differences between the learned network (LN) and the ground-truth network (GN). Specifically, *C*_*a*_ represents the number of connections accidentally added to LN, *C*_*s*_ denotes the number of same connections in LN and GN, and TC is the total number of the connections in GN.

F-measure is a harmonic mean of Precision and Recall, so it can be used to evaluate the overall performance of connections [32]. It is defined as:

$$\begin{array}{c} F_{c}=\frac{2*Precision_{c}*Recall_{c}}{Precision_{c}+Recall_{c}}. \end{array}$$ (4)

Similarly, direciton’s Precision and Recall can be defined as follows:

$$\begin{array}{c} Precision_{d}=\frac{D_{s}}{D_{w}+D_{a}+D_{s}} \end{array}$$ (5)

and

$$\begin{array}{c} Recall_{d}=\frac{D_{s}}{TD}, \end{array}$$ (6)

where *D*_*s*_, *D*_*w*_, *D*_*a*_ are used to denote the direction differences between LN and GN. Specifically, *D*_*s*_ represents the number of same arcs in LN and GN, *D*_*w*_ represents the number of arcs in LN whose connections are the same as those of GN and directions are different from the corresponding ones in GN. *D*_*a*_ shows the number of extra arcs in LN due to *C*_*a*_ connections newly added. Moreover, TD is the total number of the arcs (directions) in GN.

Naturally, direction’s F-measure is defined as:

$$\begin{array}{c} F_{d}=\frac{2*Precision_{d}*Recall_{d}}{Precision_{d}+Recall_{d}}. \end{array}$$ (7)

In particularly, if both *Precision*_*d*_ and *Recall*_*d*_ are zero, we think *F*_*d*_ should also be zero.

### Experimental results on various cases

The default parameter configurations for each algorithm are as follows. PC, CPC, CCD and FCI use the same parameters where *Alpha* = 0 and *Depth* = −1. The parameters of GES and iMaGES are set as *Penalty Discount* = 1.0, and *Num Patterns to Save* = 1. LiNGAM runs with *Prune Factor* = 1.0. Gen Synch runs with *m* = 10, *τ* = 2, *theiler* = 50, *and nn* = 10. Patel is performed with *binarisation* = 0.75. The parameters of Granger are set as *Alpha* = 0.05 and *max*_*lag* ∈ [1, 30]. Based on the results of the preliminary experiments, we found that AIAEC algorithm is not very sensitive to the parameters, and the parameter setting of AIAEC is shown as followings: *T* = 150, *P*_*s*_ = 0.5, *P*_*c*_ = 0.6, *P*_*m*_ = 0.4, *M* = 70, and *N* = 80. Moreover, larger T or N may be more likely to find the globally optimal solution at the expense of computation time. M is set from 0.7N to 0.9N, while *P*_*s*_, *P*_*c*_ and *P*_*m*_ usually do not need to change. Once some of the algorithms have different parameters in some different simulations, the specific parameter values are given in the corresponding tables. Moreover, AIAEC is run 10 times, and then the best, the worst, and the average results (i.e., *AIAEC*_*b*_, *AIAEC*_*w*_ and *AIAEC*_*a*_) over these 10 runs are shown, since AIAEC is a kind of random optimization method.

Results of all algorithms including PC, CPC, CCD, FCI, GES, iMaGES, LiNGAM, Gen Synch, Patel, Granger and AIAEC in terms of various evaluation metrics on all 28 simulated datasets are shown in Tables 2 to 8. For each algorithm, the number of the connections (Num. of Conn.) including the number of the added connections (*C*_*a*_) comparing to the corresponding ground-truth network and the number of the same connections (*C*_*s*_) as the corresponding ground-truth network, as well as the number of the directions (Num. of Dire.) including the number of the wrong directions (*D*_*w*_) comparing to the corresponding ground-truth network and the number of the same directions (*D*_*s*_) as the corresponding ground-truth network are displayed. In addition, the connection measurements including *Precision*_*c*_, *Recall*_*c*_, and *F*_*c*_ as well as direction measurements including *Precision*_*d*_, *Recall*_*d*_, and *F*_*d*_ are listed.

**Table 2:** Experimental results on Sim1–4 for eleven algorithms.

Data set	Algorithms	Num. of Conn.		Num. of Dire.		Connection Measurement			Direction Measurement		
		*C*_*a*_	*C*_*s*_	*D*_*w*_	*D*_*s*_	*Precision*_*c*_	*Recall*_*c*_	*F*_*c*_	*Precision*_*d*_	*Recall*_*d*_	*F*_*d*_
Sim1	PC	0	5	3	2	100%	100%	1	40%	40%	0.4
	CPC	0	5	4	1	100%	100%	1	20%	20%	0.2
	CCD	0	5	1	4	100%	100%	1	80%	80%	0.8
	FCI	0	5	3	2	100%	100%	1	40%	40%	0.4
	GES	0	5	3	2	100%	100%	1	40%	40%	0.4
	iMaGES	0	5	1	4	100%	100%	1	80%	80%	0.8
	LiNGAM	1	5	0	5	83%	100%	0.91	83%	100%	0.91
	Gen Synch	0	5	2	3	100%	100%	1	60%	60%	0.6
	Patel	0	5	1	4	100%	100%	1	80%	80%	0.8
	Granger	0	5	0	5	100%	100%	1	100%	100%	1
	*AIAEC*_*b*_	0	5	0	5	100%	100%	1	100%	100%	1
	*AIAEC*_*w*_	0	5	0	5	100%	100%	1	100%	100%	1
	*AIAEC*_*a*_	0	5	0	5	100%	100%	1	100%	100%	1
Sim2	PC	0	10	3	7	100%	91%	0.95	70%	64%	0.67
	CPC	0	11	6	5	100%	100%	1	45%	45%	0.45
	CCD	0	11	4	7	100%	100%	1	64%	64%	0.64
	FCI	0	11	4	7	100%	100%	1	64%	64%	0.64
	GES	0	11	4	7	100%	100%	1	64%	64%	0.64
	iMaGES	0	11	2	9	100%	100%	1	82%	82%	0.82
	LiNGAM	1	11	1	10	92%	100%	0.96	83%	91%	0.87
	Gen Synch	0	11	2	9	100%	100%	1	82%	82%	0.82
	Patel	0	11	2	9	100%	100%	1	82%	82%	0.82
	Granger	0	11	4	7	100%	100%	1	64%	64%	0.64
	*AIAEC*_*b*_	0	11	0	11	100%	100%	1	100%	100%	1
	*AIAEC*_*w*_	0	11	2	9	100%	100%	1	82%	82%	0.82
	*AIAEC*_*a*_	0	11	1	10	100%	100%	1	91%	91%	0.91
Sim3	PC	0	17	5	12	100%	94%	0.97	71%	67%	0.69
	CPC	0	18	6	12	100%	100%	1	67%	67%	0.67
	CCD	0	18	5	13	100%	100%	1	72%	72%	0.72
	FCI	0	18	5	13	100%	100%	1	72%	72%	0.72
	GES^1^	0	18	9	9	100%	100%	1	50%	50%	0.5
	iMaGES^2^	0	18	8	10	100%	100%	1	56%	56%	0.56
	LiNGAM^3^	2	18	2	16	90%	100%	0.95	80%	89%	0.84
	Gen Synch	0	18	2	16	100%	100%	1	89%	89%	0.89
	Patel	0	18	4	16	100%	100%	1	80%	89%	0.84
	Granger	1	18	6	12	95%	100%	0.97	63%	67%	0.65
	*AIAEC*_*b*_	0	18	2	16	100%	100%	1	89%	89%	0.89
	*AIAEC*_*w*_	0	18	4	14	100%	100%	1	78%	78%	0.78
	*AIAEC*_*a*_	0	18	2.8	15.2	100%	100%	1	84%	84%	0.84
Sim4	PC	0	60	15	45	100%	98%	0.99	75%	74%	0.74
	CPC	0	61	16	45	100%	100%	1	74%	74%	0.74
	CCD	0	61	24	37	100%	100%	1	61%	61%	0.61
	FCI	0	61	24	37	100%	100%	1	61%	61%	0.61
	GES^4^	0	61	25	36	100%	100%	1	59%	59%	0.59
	iMaGES^5^	0	61	24	37	100%	100%	1	61%	61%	0.61
	LiNGAM^6^	2	60	18	42	97%	98%	0.98	68%	69%	0.68
	Gen Synch	0	61	13	48	100%	100%	1	79%	79%	0.79
	Patel	0	61	14	47	100%	100%	1	77%	77%	0.77
	Granger	6	61	24	37	91%	100%	0.95	55%	61%	0.58
	*AIAEC*_*b*_	0	61	12	49	100%	100%	1	80%	80%	0.8
	*AIAEC*_*w*_	0	61	16	45	100%	100%	1	74%	74%	0.74
	*AIAEC*_*a*_	0	61	15.2	45.8	100%	100%	1	75%	75%	0.75

^1^ The parameters of GES: *Penalty Discount* = 7.0, *Num Patterns to Save* = 1.

^2^ The parameters of iMaGES: *Penalty Discount* = 7.0, *Num Patterns to Save* = 1.

^3^ The parameters of LiNGAM: *Prune Factor* = 3.0.

^4^ The parameters of GES: *Penalty Discount* = 9.0, *Num Patterns to Save* = 1.

^5^ The parameters of iMaGES: *Penalty Discount* = 9.0, *Num Patterns to Save* = 1.

^6^ The parameters of LiNGAM: *Prune Factor* = 4.0.

**Table 3:** Experimental results on Sim5–8 for eleven algorithms.

Data set	Algorithms	Num. of Conn.		Num. of Dire.		Connection Measurement			Direction Measurement		
		*C*_*a*_	*C*_*s*_	*D*_*w*_	*D*_*s*_	*Precision*_*c*_	*Recall*_*c*_	*F*_*c*_	*Precision*_*d*_	*Recall*_*d*_	*F*_*d*_
Sim5	PC	1	5	3	2	83%	100%	0.91	33%	40%	0.36
	CPC	1	5	3	2	83%	100%	0.91	33%	40%	0.36
	CCD	1	5	3	2	83%	100%	0.91	33%	40%	0.36
	FCI	1	5	3	2	83%	100%	0.91	33%	40%	0.36
	GES	0	5	1	4	100%	100%	1	80%	80%	0.8
	iMaGES	0	5	2	3	100%	100%	1	60%	60%	0.6
	LiNGAM^1^	0	5	0	5	100%	100%	1	100%	100%	1
	Gen Synch	0	5	0	5	100%	100%	1	100%	100%	1
	Patel	0	5	0	5	100%	100%	1	100%	100%	1
	Granger	0	5	2	3	100%	100%	1	60%	60%	0.6
	*AIAEC*_*b*_	0	5	0	5	100%	100%	1	100%	100%	1
	*AIAEC*_*w*_	0	5	0	5	100%	100%	1	100%	100%	1
	*AIAEC*_*a*_	0	5	0	5	100%	100%	1	100%	100%	1
Sim6	PC	2	11	4	7	85%	100%	0.92	54%	64%	0.58
	CPC	2	11	3	8	85%	100%	0.92	62%	73%	0.67
	CCD	2	11	5	6	85%	100%	0.92	46%	55%	0.5
	FCI	1	11	4	7	92%	100%	0.96	58%	64%	0.61
	GES	0	11	4	7	100%	100%	1	64%	64%	0.64
	iMaGES	0	11	3	8	100%	100%	1	73%	73%	0.73
	LiNGAM^1^	0	11	0	11	100%	100%	1	100%	100%	1
	Gen Synch	0	11	1	10	100%	100%	1	91%	91%	0.91
	Patel	0	11	1	10	100%	100%	1	91%	91%	0.91
	Granger	0	11	4	7	100%	100%	1	64%	64%	0.64
	*AIAEC*_*b*_	0	11	0	11	100%	100%	1	100%	100%	1
	*AIAEC*_*w*_	0	11	2	9	100%	100%	1	82%	82%	0.82
	*AIAEC*_*a*_	0	11	0.8	10.2	100%	100%	1	93%	93%	0.93
Sim7	PC	2	5	1	4	71%	100%	0.83	57%	80%	0.67
	CPC	2	5	1	4	71%	100%	0.83	57%	80%	0.67
	CCD	2	5	3	2	71%	100%	0.83	29%	40%	0.33
	FCI	2	5	2	3	71%	100%	0.83	43%	60%	0.5
	GES^2^	0	5	1	4	100%	100%	1	80%	80%	0.8
	iMaGES^3^	0	5	1	4	100%	100%	1	80%	80%	0.8
	LiNGAM^4^	0	5	0	5	100%	100%	1	100%	100%	1
	Gen Synch	0	5	0	5	100%	100%	1	100%	100%	1
	Patel	0	5	0	5	100%	100%	1	100%	100%	1
	Granger	0	5	1	4	100%	100%	1	80%	80%	0.8
	*AIAEC*_*b*_	0	5	0	5	100%	100%	1	100%	100%	1
	*AIAEC*_*w*_	0	5	0	5	100%	100%	1	100%	100%	1
	*AIAEC*_*a*_	0	5	0	5	100%	100%	1	100%	100%	1
Sim8	PC	2	5	4	1	71%	100%	0.83	14%	20%	0.17
	CPC	2	5	5	0	71%	100%	0.83	0%	0%	0
	CCD	2	5	3	2	71%	100%	0.83	29%	40%	0.33
	FCI	2	5	3	2	71%	100%	0.83	29%	40%	0.33
	GES	1	5	2	3	83%	100%	0.91	50%	60%	0.55
	iMaGES	1	5	3	2	83%	100%	0.91	33%	40%	0.36
	LiNGAM^5^	0	5	1	4	100%	100%	1	80%	80%	0.8
	Gen Synch	2	5	2	3	71%	100%	0.83	43%	60%	0.5
	Patel	2	5	1	4	71%	100%	0.83	57%	80%	0.67
	Granger	1	5	3	2	83%	100%	0.91	33%	40%	0.36
	*AIAEC*_*b*_	0	5	0	5	100%	100%	1	100%	100%	1
	*AIAEC*_*w*_	0	5	3	2	100%	100%	1	40%	40%	0.4
	*AIAEC*_*a*_	0	5	1.2	3.8	100%	100%	1	76%	76%	0.76

^1^ The parameters of LiNGAM: *Prune Factor* = 4.0.

^2^ The parameters of GES: *Penalty Discount* = 9.0, *Num Patterns to Save* = 1.

^3^ The parameters of iMaGES: *Penalty Discount* = 9.0, *Num Patterns to Save* = 1.

^4^ The parameters of LiNGAM: *Prune Factor* = 6.0.

^5^ The parameters of LiNGAM: *Prune Factor* = 3.0.

**Table 4:** Experimental results on Sim9–12 for eleven algorithms.

Data set	Algorithms	Num. of Conn.		Num. of Dire.		Connection Measurement			Direction Measurement		
		*C*_*a*_	*C*_*s*_	*D*_*w*_	*D*_*s*_	*Precision*_*c*_	*Recall*_*c*_	*F*_*c*_	*Precision*_*d*_	*Recall*_*d*_	*F*_*d*_
Sim9	PC	3	5	4	1	63%	100%	0.77	13%	20%	0.15
	CPC	3	5	3	2	63%	100%	0.77	25%	40%	0.31
	CCD	3	5	3	2	63%	100%	0.77	25%	40%	0.31
	FCI	3	5	3	2	63%	100%	0.77	25%	40%	0.31
	GES	3	5	2	3	63%	100%	0.77	38%	60%	0.46
	iMaGES	3	5	2	3	63%	100%	0.77	38%	60%	0.46
	LiNGAM^1^	0	5	1	4	100%	100%	1	80%	80%	0.8
	Gen Synch	3	5	1	4	63%	100%	0.77	50%	80%	0.62
	Patel	2	5	0	5	71%	100%	0.83	71%	100%	0.83
	Granger	1	5	2	3	83%	100%	0.91	50%	60%	0.55
	*AIAEC*_*b*_	0	5	0	5	100%	100%	1	100%	100%	1
	*AIAEC*_*w*_	0	5	2	3	100%	100%	1	60%	60%	0.6
	*AIAEC*_*a*_	0	5	0.8	4.2	100%	100%	1	84%	84%	0.84
Sim10	PC	0	5	4	1	100%	100%	1	20%	20%	0.2
	CPC	0	5	4	1	100%	100%	1	20%	20%	0.2
	CCD	0	5	4	1	100%	100%	1	20%	20%	0.2
	FCI	0	5	5	0	100%	100%	1	0%	0%	0
	GES	0	5	1	4	100%	100%	1	80%	80%	0.8
	iMaGES	0	5	1	4	100%	100%	1	80%	80%	0.8
	LiNGAM	1	5	0	5	83%	100%	0.91	83%	100%	0.91
	Gen Synch	0	5	0	5	100%	100%	1	100%	100%	1
	Patel	0	5	1	4	100%	100%	1	80%	80%	0.8
	Granger	0	5	0	5	100%	100%	1	100%	100%	1
	*AIAEC*_*b*_	0	5	0	5	100%	100%	1	100%	100%	1
	*AIAEC*_*w*_	0	5	0	5	100%	100%	1	100%	100%	1
	*AIAEC*_*a*_	0	5	0	5	100%	100%	1	100%	100%	1
Sim11	PC	2	6	2	4	75%	55%	0.63	50%	36%	0.42
	CPC	7	11	5	6	61%	100%	0.76	33%	55%	0.41
	CCD	7	11	2	9	61%	100%	0.76	50%	82%	0.62
	FCI	7	11	7	4	61%	100%	0.76	22%	36%	0.28
	GES^2^	9	11	6	5	55%	100%	0.71	25%	45%	0.32
	iMaGES^3^	9	11	5	6	55%	100%	0.71	30%	55%	0.39
	LiNGAM^1^	11	11	1	10	50%	100%	0.67	45%	91%	0.61
	Gen Synch	6	11	2	9	65%	100%	0.79	53%	82%	0.64
	Patel	5	11	1	10	69%	100%	0.81	63%	91%	0.74
	Granger	4	11	4	7	73%	100%	0.85	47%	64%	0.54
	*AIAEC*_*b*_	4	11	2	9	73%	100%	0.85	60%	82%	0.69
	*AIAEC*_*w*_	7	11	4	7	61%	100%	0.76	39%	64%	0.48
	*AIAEC*_*a*_	5.6	11	2.8	8.2	66%	100%	0.80	49%	75%	0.59
Sim12	PC	0	10	3	7	100%	91%	0.95	70%	64%	0.67
	CPC	0	11	6	5	100%	100%	1	45%	45%	0.45
	CCD	0	11	4	7	100%	100%	1	64%	64%	0.64
	FCI	0	11	4	7	100%	100%	1	64%	64%	0.64
	GES	0	11	2	9	100%	100%	1	82%	82%	0.82
	iMaGES	0	11	2	9	100%	100%	1	82%	82%	0.82
	LiNGAM	0	11	2	9	100%	100%	1	82%	82%	0.82
	Gen Synch	0	11	2	9	100%	100%	1	82%	82%	0.82
	Patel	0	11	1	10	100%	100%	1	91%	91%	0.91
	Granger	0	11	1	10	100%	100%	1	91%	91%	0.91
	*AIAEC*_*b*_	0	11	0	11	100%	100%	1	100%	100%	1
	*AIAEC*_*w*_	0	11	2	9	100%	100%	1	82%	82%	0.82
	*AIAEC*_*a*_	0	11	1.2	9.8	100%	100%	1	89%	89%	0.89

^1^ The parameters of LiNGAM: *Prune Factor* = 3.0.

^2^ The parameters of GES: *Penalty Discount* = 6.0, *Num Patterns to Save* = 1.

^3^ The parameters of iMaGES: *Penalty Discount* = 6.0, *Num Patterns to Save* = 1.

**Table 5:** Experimental results on Sim13–16 for eleven algorithms.

Data set	Algorithms	Num. of Conn.		Num. of Dire.		Connection Measurement			Direction Measurement		
		*C*_*a*_	*C*_*s*_	*D*_*w*_	*D*_*s*_	*Precision*_*c*_	*Recall*_*c*_	*F*_*c*_	*Precision*_*d*_	*Recall*_*d*_	*F*_*d*_
Sim13	PC	0	3	1	2	100%	60%	0.75	67%	40%	0.5
	CPC	0	3	3	0	100%	60%	0.75	0%	0%	0
	CCD	0	3	1	2	100%	60%	0.75	67%	40%	0.5
	FCI	0	3	1	2	100%	60%	0.75	67%	40%	0.5
	GES	1	4	0	4	80%	80%	0.8	80%	80%	0.8
	iMaGES	1	4	0	4	80%	80%	0.8	80%	80%	0.8
	LiNGAM	2	3	1	2	60%	60%	0.6	40%	40%	0.4
	Gen Synch	0	3	2	1	100%	60%	0.75	33%	20%	0.25
	Patel	0	3	1	2	100%	60%	0.75	67%	40%	0.5
	Granger	1	3	2	1	75%	60%	0.67	25%	20%	0.22
	*AIAEC*_*b*_	0	5	1	4	100%	100%	1	80%	80%	0.80
	*AIAEC*_*w*_	0	5	3	2	100%	100%	1	40%	40%	0.4
	*AIAEC*_*a*_	0	5	1.8	3.2	100%	100%	1	64%	64%	0.64
Sim14	PC	0	5	5	0	100%	100%	1	0%	0%	0
	CPC	0	5	5	0	100%	100%	1	0%	0%	0
	CCD	0	5	4	1	100%	100%	1	20%	20%	0.2
	FCI	0	5	5	0	100%	100%	1	0%	0%	0
	GES^1^	0	5	1	4	100%	100%	1	80%	80%	0.8
	iMaGES^2^	0	5	1	4	100%	100%	1	80%	80%	0.8
	LiNGAM^3^	0	5	1	4	100%	100%	1	80%	80%	0.8
	Gen Synch	0	5	0	5	100%	100%	1	100%	100%	1
	Patel	0	5	3	2	100%	100%	1	50%	60%	0.55
	Granger	1	5	1	4	83%	100%	0.91	67%	80%	0.73
	*AIAEC*_*b*_	0	5	1	4	100%	100%	1	80%	80%	0.8
	*AIAEC*_*w*_	0	5	3	2	100%	100%	1	40%	40%	0.4
	*AIAEC*_*a*_	0	5	2.4	2.6	100%	100%	1	52%	52%	0.52
Sim15	PC	1	5	3	2	83%	100%	0.91	33%	40%	0.36
	CPC	1	5	3	2	83%	100%	0.91	33%	40%	0.36
	CCD	1	5	3	2	83%	100%	0.91	33%	40%	0.36
	FCI	0	5	3	2	100%	100%	1	40%	40%	0.4
	GES^1^	0	5	2	3	100%	100%	1	60%	60%	0.6
	iMaGES^2^	0	5	1	4	100%	100%	1	80%	80%	0.8
	LiNGAM^3^	1	5	2	3	83%	100%	0.91	50%	60%	0.55
	Gen Synch	3	5	2	3	63%	100%	0.77	38%	60%	0.46
	Patel	2	5	0	5	71%	100%	0.83	71%	100%	0.83
	Granger	3	5	2	3	63%	100%	0.77	38%	60%	0.46
	*AIAEC*_*b*_	0	5	0	5	100%	100%	1	100%	100%	1
	*AIAEC*_*w*_	0	5	1	4	100%	100%	1	80%	80%	0.8
	*AIAEC*_*a*_	0	5	0.2	4.8	100%	100%	1	96%	96%	0.96
Sim16	PC	0	7	4	3	100%	100%	1	43%	43%	0.43
	CPC^4^	1	7	4	3	88%	100%	0.93	38%	43%	0.4
	CCD	0	7	4	3	100%	100%	1	43%	43%	0.43
	FCI	0	7	4	3	100%	100%	1	43%	43%	0.43
	GES	0	7	4	3	100%	100%	1	43%	43%	0.43
	iMaGES	0	7	4	3	100%	100%	1	43%	43%	0.43
	LiNGAM	1	7	2	5	88%	100%	0.93	63%	71%	0.67
	Gen Synch	1	7	2	5	88%	100%	0.93	63%	71%	0.67
	Patel	1	7	0	7	88%	100%	0.93	88%	100%	0.93
	Granger	1	7	4	3	88%	100%	0.93	38%	43%	0.4
	*AIAEC*_*b*_	0	7	2	5	100%	100%	1	71%	71%	0.71
	*AIAEC*_*w*_	0	7	4	3	100%	100%	1	43%	43%	0.43
	*AIAEC*_*a*_	0	7	2.8	4.2	100%	100%	1	60%	60%	0.6

^1^ The parameters of GES: *Penalty Discount* = 6.0, *Num Patterns to Save* = 1.

^2^ The parameters of iMaGES: *Penalty Discount* = 6.0, *Num Patterns to Save* = 1.

^3^ The parameters of LiNGAM: *Prune Factor* = 3.0.

^4^ The parameters of CPC: *Alpha* = 0.01, *Depth* = −1.

**Table 6:** Experimental results on Sim17–20 for eleven algorithms.

Data set	Algorithms	Num. of Conn.		Num. of Dire.		Connection Measurement			Direction Measurement		
		*C*_*a*_	*C*_*s*_	*D*_*w*_	*D*_*s*_	*Precision*_*c*_	*Recall*_*c*_	*F*_*c*_	*Precision*_*d*_	*Recall*_*d*_	*F*_*d*_
Sim17	PC	0	10	3	7	100%	91%	0.95	70%	64%	0.67
	CPC	0	11	4	7	100%	100%	1	64%	64%	0.64
	CCD	0	11	4	7	100%	100%	1	64%	64%	0.64
	FCI	0	11	4	7	100%	100%	1	64%	64%	0.64
	GES	0	11	2	9	100%	100%	1	82%	82%	0.82
	iMaGES	0	11	2	9	100%	100%	1	82%	82%	0.82
	LiNGAM^1^	0	11	1	10	100%	100%	1	91%	91%	0.91
	Gen Synch	0	11	0	11	100%	100%	1	100%	100%	1
	Patel	0	11	1	10	100%	100%	1	91%	91%	0.91
	Granger	3	11	2	9	79%	100%	0.88	64%	82%	0.72
	*AIAEC*_*b*_	0	11	0	11	100%	100%	1	100%	100%	1
	*AIAEC*_*w*_	0	11	1	10	100%	100%	1	91%	91%	0.91
	*AIAEC*_*a*_	0	11	0.2	10.8	100%	100%	1	98%	98%	0.98
Sim18	PC	0	5	3	2	100%	100%	1	40%	40%	0.4
	CPC	0	5	4	1	100%	100%	1	20%	20%	0.2
	CCD	0	5	1	4	100%	100%	1	80%	80%	0.8
	FCI	0	5	4	1	100%	100%	1	20%	20%	0.2
	GES	0	5	2	3	100%	100%	1	60%	60%	0.6
	iMaGES	0	5	1	4	100%	100%	1	80%	80%	0.8
	LiNGAM^1^	0	5	0	5	100%	100%	1	100%	100%	1
	Gen Synch	0	5	0	5	100%	100%	1	100%	100%	1
	Patel	0	5	1	4	100%	100%	1	80%	80%	0.8
	Granger	0	5	1	4	100%	100%	1	80%	80%	0.8
	*AIAEC*_*b*_	0	5	0	5	100%	100%	1	100%	100%	1
	*AIAEC*_*w*_	0	5	1	4	100%	100%	1	80%	80%	0.8
	*AIAEC*_*a*_	0	5	0.6	4.4	100%	100%	1	88%	88%	0.88
Sim19	PC	0	5	3	2	100%	100%	1	40%	40%	0.4
	CPC	0	5	3	2	100%	100%	1	40%	40%	0.4
	CCD	0	5	3	2	100%	100%	1	40%	40%	0.4
	FCI	0	5	3	2	100%	100%	1	40%	40%	0.4
	GES	0	5	3	2	100%	100%	1	40%	40%	0.4
	iMaGES	0	5	3	2	100%	100%	1	40%	40%	0.4
	LiNGAM^1^	0	5	0	5	100%	100%	1	100%	100%	1
	Gen Synch	0	5	0	5	100%	100%	1	100%	100%	1
	Patel	0	5	1	4	100%	100%	1	80%	80%	0.8
	Granger	2	5	1	4	71%	100%	0.83	57%	80%	0.67
	*AIAEC*_*b*_	0	5	0	5	100%	100%	1	100%	100%	1
	*AIAEC*_*w*_	0	5	0	5	100%	100%	1	100%	100%	1
	*AIAEC*_*a*_	0	5	0	5	100%	100%	1	100%	100%	1
Sim20	PC	0	5	3	2	100%	100%	1	40%	40%	0.4
	CPC	0	5	3	2	100%	100%	1	40%	40%	0.4
	CCD	0	5	3	2	100%	100%	1	40%	40%	0.4
	FCI	0	5	3	2	100%	100%	1	40%	40%	0.4
	GES	0	5	1	4	100%	100%	1	80%	80%	0.8
	iMaGES	0	5	1	4	100%	100%	1	80%	80%	0.8
	LiNGAM^1^	0	5	0	5	100%	100%	1	100%	100%	1
	Gen Synch	0	5	0	5	100%	100%	1	100%	100%	1
	Patel	0	5	1	4	100%	100%	1	80%	80%	0.8
	Granger	4	5	0	5	56%	100%	0.71	56%	100%	0.71
	*AIAEC*_*b*_	0	5	0	5	100%	100%	1	100%	100%	1
	*AIAEC*_*w*_	0	5	0	5	100%	100%	1	100%	100%	1
	*AIAEC*_*a*_	0	5	0	5	100%	100%	1	100%	100%	1

^1^ The parameters of LiNGAM: *Prune Factor* = 3.0.

**Table 7:** Experimental results on Sim21–24 for eleven algorithms.

Data set	Algorithms	Num. of Conn.		Num. of Dire.		Connection Measurement			Direction Measurement		
		*C*_*a*_	*C*_*s*_	*D*_*w*_	*D*_*s*_	*Precision*_*c*_	*Recall*_*c*_	*F*_*c*_	*Precision*_*d*_	*Recall*_*d*_	*F*_*d*_
Sim21^1^	PC	0/0	5/5	3/2	2/3	100%/100%	100%/100%	1/1	40%/60%	40%/60%	0.4/0.6
	CPC	0/0	5/5	4/4	1/1	100%/100%	100%/100%	1/1	20%/20%	20%/20%	0.2/0.2
	CCD	0/0	5/5	1/0	4/5	100%/100%	100%/100%	1/1	80%/100%	80%/100%	0.8/1
	FCI	0/0	5/5	3/3	2/2	100%/100%	100%/100%	1/1	40%/60%	40%/60%	0.4/0.6
	GES	0/0	5/5	2/0	3/5	100%/100%	100%/100%	1/1	60%/100%	60%/100%	0.6/1
	iMaGES	0/0	5/5	2/0	3/5	100%/100%	100%/100%	1/1	60%/100%	60%/100%	0.6/1
	LiNGAM	0/0	5/5	0/0	5/5	100%/100%	100%/100%	1/1	100%/100%	100%/100%	1/1
	Gen Synch	0/0	5/5	2/2	3/3	100%/100%	100%/100%	1/1	60%/60%	60%/60%	0.6/0.6
	Patel	0/0	5/5	1/1	4/4	100%/100%	100%/100%	1/1	80%/80%	80%/80%	0.8/0.8
	Granger	0/0	4/5	0/1	4/4	100%/100%	80%/100%	0.89/1	100%/80%	80%/80%	0.89/0.8
	*AIAEC*_*b*_	0/0	5/5	0/0	5/5	100%/100%	100%/100%	1/1	100%/100%	100%/100%	1/1
	*AIAEC*_*w*_	0/0	5/5	1/0	4/5	100%/100%	100%/100%	1/1	80%/100%	80%/100%	0.8/1
	*AIAEC*_*a*_	0/0	5/5	0.6/0	4.4/5	100%/100%	100%/100%	1/1	88%/100%	88%/100%	0.88/1
Sim22	PC	0	5	3	2	100%	100%	1	40%	40%	0.4
	CPC	0	5	3	2	100%	100%	1	40%	40%	0.4
	CCD	0	5	1	4	100%	100%	1	80%	80%	0.8
	FCI	0	5	3	2	100%	100%	1	40%	40%	0.4
	GES	0	5	1	4	100%	100%	1	80%	80%	0.8
	iMaGES	0	5	0	5	100%	100%	1	100%	100%	1
	LiNGAM	2	5	4	1	71%	100%	0.83	14%	20%	0.17
	Gen Synch	0	5	0	5	100%	100%	1	100%	100%	1
	Patel	0	5	0	5	100%	100%	1	100%	100%	1
	Granger	0	4	2	2	100%	80%	0.89	50%	40%	0.44
	*AIAEC*_*b*_	0	5	0	5	100%	100%	1	100%	100%	1
	*AIAEC*_*w*_	0	5	0	5	100%	100%	1	100%	100%	1
	*AIAEC*_*a*_	0	5	0	5	100%	100%	1	100%	100%	1
Sim23	PC	1	5	3	2	83%	100%	0.91	33%	40%	0.36
	CPC	1	5	3	2	83%	100%	0.91	33%	40%	0.36
	CCD	1	5	4	1	83%	100%	0.91	17%	20%	0.18
	FCI	1	5	5	0	83%	100%	0.91	0%	0%	0
	GES^2^	2	5	2	3	71%	100%	0.83	43%	60%	0.5
	iMaGES^3^	2	5	1	4	71%	100%	0.83	57%	80%	0.67
	LiNGAM	1	5	3	2	83%	100%	0.91	33%	40%	0.36
	Gen Synch	2	5	0	5	71%	100%	0.83	71%	100%	0.83
	Patel	3	5	1	4	63%	100%	0.77	50%	80%	0.62
	Granger	3	5	2	3	63%	100%	0.77	38%	60%	0.46
	*AIAEC*_*b*_	2	5	1	4	71%	100%	0.83	57%	80%	0.67
	*AIAEC*_*w*_	2	5	1	4	71%	100%	0.83	57%	80%	0.67
	*AIAEC*_*a*_	2	5	1	4	71%	100%	0.83	57%	80%	0.67
Sim24	PC	4	5	4	1	56%	100%	0.71	11%	20%	0.14
	CPC	4	5	5	0	56%	100%	0.71	0%	0%	0
	CCD	4	5	5	0	56%	100%	0.71	0%	0%	0
	FCI	4	5	4	1	56%	100%	0.71	11%	20%	0.14
	GES^2^	3	5	1	4	63%	100%	0.77	50%	80%	0.62
	iMaGES^3^	3	5	2	3	63%	100%	0.77	38%	60%	0.46
	LiNGAM	2	5	2	3	71%	100%	0.83	43%	60%	0.5
	Gen Synch	4	5	2	3	56%	100%	0.71	33%	60%	0.43
	Patel	3	5	1	4	63%	100%	0.77	50%	80%	0.62
	Granger	4	5	1	4	56%	100%	0.71	44%	80%	0.57
	*AIAEC*_*b*_	1	5	1	4	83%	100%	0.91	67%	80%	0.73
	*AIAEC*_*w*_	2	5	1	4	71%	100%	0.83	57%	80%	0.67
	*AIAEC*_*a*_	1.8	5	1	4	74%	100%	0.85	59%	80%	0.68

^1^ Sim21 shows the results of the two groups, shown as group1/group2.

^2^ The parameters of GES: *Penalty Discount* = 6.0, *Num Patterns to Save* = 1.

^3^ The parameters of iMaGES: *Penalty Discount* = 6.0, *Num Patterns to Save* = 1.

**Table 8:** Experimental results on Sim25–28 for eleven algorithms.

Data set	Algorithms	Num. of Conn.		Num. of Dire.		Connection Measurement			Direction Measurement		
		*C*_*a*_	*C*_*s*_	*D*_*w*_	*D*_*s*_	*Precision*_*c*_	*Recall*_*c*_	*F*_*c*_	*Precision*_*d*_	*Recall*_*d*_	*F*_*d*_
Sim25	PC	0	5	3	2	100%	100%	1	40%	40%	0.4
	CPC	0	5	4	1	100%	100%	1	20%	20%	0.2
	CCD	0	5	1	4	100%	100%	1	80%	80%	0.8
	FCI	0	5	3	2	100%	100%	1	40%	40%	0.4
	GES	0	5	0	5	100%	100%	1	100%	100%	1
	iMaGES	0	5	1	4	100%	100%	1	80%	80%	0.8
	LiNGAM	1	5	1	4	83%	100%	0.91	67%	80%	0.73
	Gen Synch	0	5	1	4	100%	100%	1	80%	80%	0.8
	Patel	0	5	1	4	100%	100%	1	80%	80%	0.8
	Granger	0	5	2	3	100%	100%	1	60%	60%	0.60
	*AIAEC*_*b*_	0	5	0	5	100%	100%	1	100%	100%	1
	*AIAEC*_*w*_	0	5	1	4	100%	100%	1	80%	80%	0.8
	*AIAEC*_*a*_	0	5	0.1	4.9	100%	100%	1	98%	98%	0.98
Sim26	PC	0	5	2	3	100%	100%	1	60%	60%	0.6
	CPC	0	5	2	3	100%	100%	1	60%	60%	0.6
	CCD	0	5	2	3	100%	100%	1	60%	60%	0.6
	FCI	0	5	2	3	100%	100%	1	60%	60%	0.6
	GES	0	5	1	4	100%	100%	1	80%	80%	0.8
	iMaGES	0	5	2	3	100%	100%	1	60%	60%	0.6
	LiNGAM^1^	0	5	2	3	100%	100%	1	60%	60%	0.6
	Gen Synch	0	5	2	3	100%	100%	1	60%	60%	0.6
	Patel	0	5	1	4	100%	100%	1	80%	80%	0.8
	Granger	2	5	3	2	71%	100%	0.83	29%	40%	0.33
	*AIAEC*_*b*_	0	5	0	5	100%	100%	1	100%	100%	1
	*AIAEC*_*w*_	0	5	1	4	100%	100%	1	80%	80%	0.8
	*AIAEC*_*a*_	0	5	0.2	4.8	100%	100%	1	96%	96%	0.96
Sim27	PC	0	5	3	2	100%	100%	1	40%	40%	0.4
	CPC	0	5	4	1	100%	100%	1	20%	20%	0.2
	CCD	0	5	1	4	100%	100%	1	80%	80%	0.8
	FCI	0	5	3	2	100%	100%	1	40%	40%	0.4
	GES	0	5	1	4	100%	100%	1	80%	80%	0.8
	iMaGES	0	5	1	4	100%	100%	1	80%	80%	0.8
	LiNGAM	0	5	0	5	100%	100%	1	100%	100%	1
	Gen Synch	0	5	1	4	100%	100%	1	80%	80%	0.8
	Patel	0	5	1	4	100%	100%	1	80%	80%	0.8
	Granger	3	5	3	2	63%	100%	0.77	25%	40%	0.31
	*AIAEC*_*b*_	0	5	0	5	100%	100%	1	100%	100%	1
	*AIAEC*_*w*_	0	5	0	5	100%	100%	1	100%	100%	1
	*AIAEC*_*a*_	0	5	0	5	100%	100%	1	100%	100%	1
Sim28	PC	0	5	3	2	100%	100%	1	40%	40%	0.4
	CPC	0	5	3	2	100%	100%	1	40%	40%	0.4
	CCD	0	5	1	4	100%	100%	1	80%	80%	0.8
	FCI	0	5	3	2	100%	100%	1	40%	40%	0.4
	GES	0	5	1	4	100%	100%	1	80%	80%	0.8
	iMaGES	0	5	1	4	100%	100%	1	80%	80%	0.8
	LiNGAM	0	5	0	5	100%	100%	1	100%	100%	1
	Gen Synch	0	5	0	5	100%	100%	1	100%	100%	1
	Patel	0	5	1	4	100%	100%	1	80%	80%	0.8
	Granger	0	4	1	3	100%	80%	0.89	75%	60%	0.67
	*AIAEC*_*b*_	0	5	0	5	100%	100%	1	100%	100%	1
	*AIAEC*_*w*_	0	5	0	5	100%	100%	1	100%	100%	1
	*AIAEC*_*a*_	0	5	0	5	100%	100%	1	100%	100%	1

^1^ The parameters of LiNGAM: *Prune Factor* = 3.0.

#### Situation 1: Factor of network node number

The detailed comparison results of Sim1, Sim2, Sim3 and Sim4 datasets are shown in Table 2. The four simulated datasets have the same test conditions, i.e., 10 min fMRI sessions for each subject, 50 subjects, TR = 3 s, final added noise of 1%, and HRF variability of ±0.5s. In this situation, the only difference is the number of nodes. That is, the numbers of nodes in Sim1, Sim2, Sim3 and Sim4 datasets are 5, 10, 15 and 50, respectively. Following the chain of Sim1-Sim2-Sim3-Sim4 (node number increasing), it was found that AIAEC has a little decrease of *F*_*d*_, while still has comparable or better performance to other algorithms. In Sim1, all algorithms except LiNGAM perform excellent on identifying network connections. As for connection directions, AIAEC and Granger have the best performance, and directions identified by them are entirely consistent with those of the ground-truth network. With the 10 nodes in Sim2, AIAEC obtains 5 times the best results over 10 running. *AIAEC*_*b*_ and *AIAEC*_*a*_ get the highest *F*_*d*_, and *AIAEC*_*w*_ get the same performance with that of iMaGES, Gen Synch and Patel. When the number of nodes increases to 15 or more, none of the methods in Sim3 and Sim4 can correctly identify all directions. For the 15 nodes in Sim3, *AIAEC*_*b*_ and Gen Synch perform best. The average performance of AIAEC (*AIAEC*_*a*_) also perform well, which is the same as that of LiNGAM and Patel. For the 50 nodes in Sim4, *AIAEC*_*b*_ has the best performance among all algorithms. Though the *F*_*d*_ values of *AIAEC*_*w*_ and *AIAEC*_*a*_ are inferior to Gen Synch and Patel, they are still equal to or better than the other eight algorithms. From overall perspective, the increase of the node number will affect to a certain extent the performance of AIAEC and some other algorithms, however, AIAEC still has good performance.

#### Situation 2: Factor of session durations

The experimental results of Sim5, Sim6 and Sim7 are shown in Table 3. Sim5 and Sim7 have the same conditions as Sim1 except for different session lengths. Specifically, Sim5 has 60-min sessions while Sim7 has 250-min sessions. Sim6 has the same conditions as Sim2, but contains 60-min sessions. Sim25 and Sim26 also have the same conditions with Sim1, the only difference is that Sim25 contains 5-min sessions while Sim26 has 2.5-min sessions. Following the chain of Sim26-Sim25-Sim1-Sim5-Sim7 (i.e., the session duration is increasing from 2.5 min, to 5 min, 10 min, 60 min, 250 min) and the chain of Sim2-Sim6 (the session duration is increasing from 10 min to 60 min), it was found that AIAEC can get the stable solution of the effective connectivity from short time to long time, while other algorithms have an obvious setback when the session length decreases. From the comparison between Tables 2 and 3, we can clearly see that most of algorithms have improved on direction measurement as the fMRI session length increasing. These results are consistent with the research of Smith et at. (2011). Compared with Sim1, AIAEC still performs well at estimating directionality in Sim5, all of which get the *F*_*d*_ of 1. Moreover, LiNGAM has improved a lot in *F*_*d*_, which also verifies the view mentioned in Simth et al. (2011): the increased number of timepoints helps temporal ICA function well in LiNGAM. Compared with Sim2, Sim6 also gets the similar results with Sim5, where the *F*_*d*_ value of *AIAEC*_*a*_ has increased to 0.93 from 0.91. In Sim7, AIAEC still performs well, and some other algorithms improved. Moreover, the similar pattern is also verified by partial results in Table 8, where in Sim25, it was found that AIAEC performs well when the number of data points is smaller (recording length is shorter) while some algorithms’ performance obviously declines. When the session durations reduce to 2.5-min in Sim26, AIAEC still maintains the accurate direction judgments though some algorithms (e.g., LiNGAM and Granger) have a significant decrease on direction metrics. That is, AIAEC has a better performance than other algorithms in the cases of shorter session, which will be very beneficial to the real fMRI data research because people usually can not get a very long fMRI data in many cases, especially for subjects of brain diseases. In other words, the shorter session lengths lead to most algorithms performing worse, while AIAEC always performs well whenever the session durations are long or short.

#### Situation 3: Factor of the shared inputs

Sim8 and Sim9 introduce the shared inputs in the 5 node simulations, which means that the external inputs are mixed into the network. These external inputs can be thought of as neuronal “noise” in these simulations. Besides the shared inputs, Sim8 has the same conditions with Sim1 while Sim9 has the same conditions with Sim7. The experimental results on these two simulations are shown in Tables 3 and 4. Compared to corresponding results in Sim1 and Sim7, the experimental results in Sim8 and Sim9 show that: the shared inputs seriously affect the performance of AIA and other algorithms whether on network connections or connection directions. In Sim8, *AIAEC*_*b*_, *AIAEC*_*a*_ and LiGAM perform well, *AIAEC*_*w*_ is not quite well. In Sim9, other methods except for AIAEC, LiNGAM and Patel have obvious drawback on inferring network connections and directions. From the comparison results, we can see external inputs affect the performance of most algorithms, while AIAEC has obvious advantages on network connections and directions in these two simulations.

#### Situation 4: Factor of global mean confound

Sim10 shows the situation with global mean confound in Sim1. The global mean confound means to add the same random timeseries to all nodes’ BOLD timeseries. The comparison results between Sim1 and Sim10 show that the global mean confound has no significant impact on AIAEC performance, where *AIAEC*_*b*_, *AIAEC*_*w*_ and *AIAEC*_*a*_ can correctly identify all the network connections and connection directions. In this simulation, Gen Synch and Granger also perform well, especially they can correctly identify directions. Compared with Sim1, PC, CCD and FCI perform worse while GES and Gen Synch perform better. So it’s not sure that global mean confound has good effects or bad effects on the different algorithms, such similar conclusion also was mentioned in [33].

#### Situation 5: Factor of bad ROIs

Sim11 and Sim12 show the situation of bad ROIs—mixing the BOLD timeseries with each other. Besides the bad ROIs, Sim11 and Sim12 have the same conditions with Sim2. However, Sim11 and Sim12 have different bad ROIs. In Sim11, each node’s timeseries are mixed in a relatively small amount of one other node’s timeseries (randomly chosen, but the same for all subjects). But in Sim12, each timeseries of interest is mixed in unrelated timeseries (achieved, for each subject, by using data from another subject). From Table 4, we find that the bad ROIs in Sim11 result into a great impact on detecting network connections and connection directions to AIA and other algorithms, while the bad ROIs in Sim12 have no obvious effect on them. In Sim11, none of algorithms perform well at estimating directionality, *AIAEC*_*b*_ is comparable with Patel which is better than other algorithms. In Sim12, all algorithms except PC correctly detected network connections, and only *AIAEC*_*b*_ can correctly identify network directions.

#### Situation 6: Factor of backwards connections

Sim13 shows the situation of backwards connections. As mentioned by Smith et al. (2011), they randomly selected half of the forwards connections in Sim1, and added a negative backwards connection of equal average strength (0.4±0.1). Compared the results with Sim1, the factor of backwards connections has no effect on identifying network connections of AIAEC while make AIAEC performs worse on identifying connection directions. In addition, other algorithms not only perform worse on identifying connection directions, but also have an obvious drawback on detecting network connections. So the factor of backwards connections affects most algorithms, and makes them perform worse.

#### Situation 7: Factor of cyclic connections

Sim14 shows the situation where there is a cyclic causality by reversing the direction of arc 1 → 5. In this simulation, the ground-truth network is changed, and is different from that of other 5 node networks. This condition is a fatal problem for many of the global network modeling approaches including most of the Bayes net methods, as it breaks the general modeling assumption implied in these approaches, i.e., there is no cycle in the graph. As shown in Table 5, the factor of cyclic connections seriously affects the performance of AIAEC on identifying connection directions. All algorithms perform well on connection metrics, and every algorithm except Granger obtains the best result. For direction metrics, most of the Bayes Net methods are fallen, while Gen Synch can correctly identify all the directions. Another interesting thing is that when the direction of arc 1 → 5 change to 5 → 1, the *AIAEC*_*b*_, GES, iMaGES are all false to identify the direction of arc 1 → 2. This may be because these methods obey the assumption that the graph has no cycle. So the factor of cyclic connections has effect on Bayes Net methods, and leads to inaccurate identification of directions.

#### Situation 8: Factor of stronger connections

Sim15 shows the situation where the strength of the network connections is increased to a mean of 0.9 instead of 0.4. Since the number of nodes 5 or 10 has no obvious effect on the performance of most algorithms, we do a comparison between Sim15 and Sim17. The two cases have the same conditions besides the strength of the network connections and number of nodes. Compared with Sim17, the factor of strong connections has no obvious effect on AIAEC. However, the increasing strength of connections leads to many approaches fall in detecting network connections, while most Bayes net methods still have excellent performances. Especially, AIAEC, FCI, GES and iMaGES correctly detect all the connections. Meanwhile, all algorithms except AIAEC perform worse at estimating directionality in Sim15 than that of in Sim17. So the factor of stronger connections has bad effect to all algorithms besides AIAEC on identifying connection directions.

#### Situation 9: Factor of more connections

Sim16 shows the situation where there are more connections in 5 nodes’ networks. Different from the ground-truth network in Sim1, the ground-truth network in Sim16 adds two arcs 2 → 4 and 3 → 5 while other conditions are the same as that of Sim1. In Sim16, more connections make AIAEC perform worse. Compared with Sim1, performance of PC, CPC, FCI, GES, Gen Synch and Patel improved, while CCD, iMaGES, LiNGAM and Granger have declined. *AIAEC*_*a*_ has the comparable performance with LiNGAM and Gen Synch which is better than other algorithms except for Patel. In conclusion, the factor of more connections has different effect on different algorithms.

#### Situation 10: Factor of HRF variability and low TR

Compared to Sim1, Sim18 has the same conditions except that HRF variability is set to 0s, Sim19 reduces the TR to 0.25s, sets the noise to 0.1% and increases the neural lag to 100ms, and Sim20 is a further version of Sim19 by removing the HRF variability. From Table 6, it was found that most of the algorithms have the similar performance as Sim1 where all algorithms can correctly detect network connections in Sim18. Moreover, LiNGAM, Gen Synch and AIAEC also perform well on identifying connection directions. In Sim19 and Sim20, LiNGAM, Gen Synch and AIAEC perform excellent, they can correctly detect all network connections and connection directions. In particular, AIAEC has a stable performance, the *F*_*d*_ values of *AIAEC*_*b*_, *AIAEC*_*w*_ and *AIAEC*_*a*_ are 1.

#### Situation 11: Factor of 2-group test

Sim21 has the same conditions as Sim1 except for 2-group test. The 2-group test in Smith et al. (2011) is to test how sensitive the different methods are at detecting changes in connection strength across different subjects. In our experiment, we make the 50 subjects into two groups. That is, the former 25 subjects with the same connection strength as that of Sim1 are divided in group1, and the latter 25 subjects with half of connection strength are divided in group2. Then all algorithms are used to test with these two groups, respectively. The results of Sim21 are shown in Table 7 which contains the test of all algorithms in the two groups. From the table we can see, AIAEC and most of the algorithms can find the changes of connection strength. With the reduction of the connection strength, most algorithms’ performances on group2 are better than those on group1. More importantly, AIAEC performs well, which is comparable to LiNGAM, and can dramatically find the changes of connection strength between the two groups.

#### Situation 12: Factor of nonstationary and stationarity connection strengths

Sim22 and Sim23 investigate the factor of nonstationarity and stationarity of connection strength between nodes. Sim23 has 5 nodes, noise of 0.1%, strong connections (mean 0.9) and reduced strength of 0.3 for all external inputs apart from node1, this situation is called stationarity connection strengths. Sim22 is the same as Sim23, except that the connection strength is modulated over time by additional random processes, and this situation is called nonstationary connection strengths. From the results, it was found AIAEC perform well in Sim22 which is the same as in Sim1, indicating that nonstationary connection strengths has no effect on AIAEC. While AIAEC has an obvious setback in Sim23 compared to Sim1, which shows the factor of stationarity connection strengths has a bad effect on AIAEC. In Sim22, most of the algorithms keep the same performance, iMaGES, Gen Synch, Patel and AIAEC perform very well, correctly identifying all directions. LiNGAM performs the worst, and its *F*_*d*_ value is only 0.17, this result is consistent with the views in Hyvärinen and Smith (2013). In their paper [34], they said that nonstationary connection strengths in Sim22 violate the basic assumption of the model employed by LiNGAM. In Sim23, no algorithm can correctly detect all connection directions. More specifically, though AIAEC is inferior to Gen Synch which performs the best in the case, it obtains the second best performance, which is comparable to iMaGES and better than other algorithms. In the two situations, we found that factor of stationarity connection strengths has a bad effect on most of the algorithms, while factor of nonstationary connection strengths has no effect on most algorithms and even improves some algorithms’ performance.

#### Situation 13: Factor of having only one stronger external input

Different from Sim15 where each node has a strong external input, Sim24 shows the situation that there is only one stronger external input. More specifically, all nodes apart from node 1 have their own external input strengths reduced from 1 to 0.1 in Sim24. Compared with Sim15, it was found that AIAEC and most algorithms become poor on performance. Though no algorithm can detect network connections entirely correctly, AIAEC performs the best. Similarly, AIAEC also performs the best on estimating directionality, the *F*_*d*_ values of *AIAEC*_*b*_, *AIAEC*_*w*_ and *AIAEC*_*a*_ are 0.73, 0.67 and 0.68, respectively. Even in the worst case, the *F*_*d*_ value of *AIAEC*_*w*_ is higher than the second best algorithms (GES and Patel) and much better than other Bayes net algorithms. In this situation, all algorithms except for GES have an obvious setback, the results show that the factor of having only one stronger external input make most algorithms perform worse.

#### Situation 14: Factor of different noises

Sim27 and Sim28 are two variations of Sim26 and Sim25, respectively, by reducing the noise to 0.1%. Comparing these results in Table 8, it is not difficult to see that the noise reduction can make a small improvement for AIA and most of the algorithms on estimating connection directions. Along with the noise reduction in Sim27 and Sim28, AIAEC can correctly identify all connection directions in all cases. It’s worth recalling that Sim26 has the worst condition compared with other three simulations. In the simulation, AIAEC still maintains the best on the performance of directionality, where the *F*_*d*_ values of *AIAEC*_*b*_, *AIAEC*_*w*_ and *AIAEC*_*a*_ are 1, 0.8 and 0.96, respectively. Even in the worst case, AIAEC is not inferior to the second best algorithms (GES and Patel) and much better than other algorithms. These results on the situation show that AIAEC has very good performances when the session is short and the noise is significant. From another aspect, it shows that high noises make algorithms perform worse.

#### Comparative network structures

**Fig 6:**
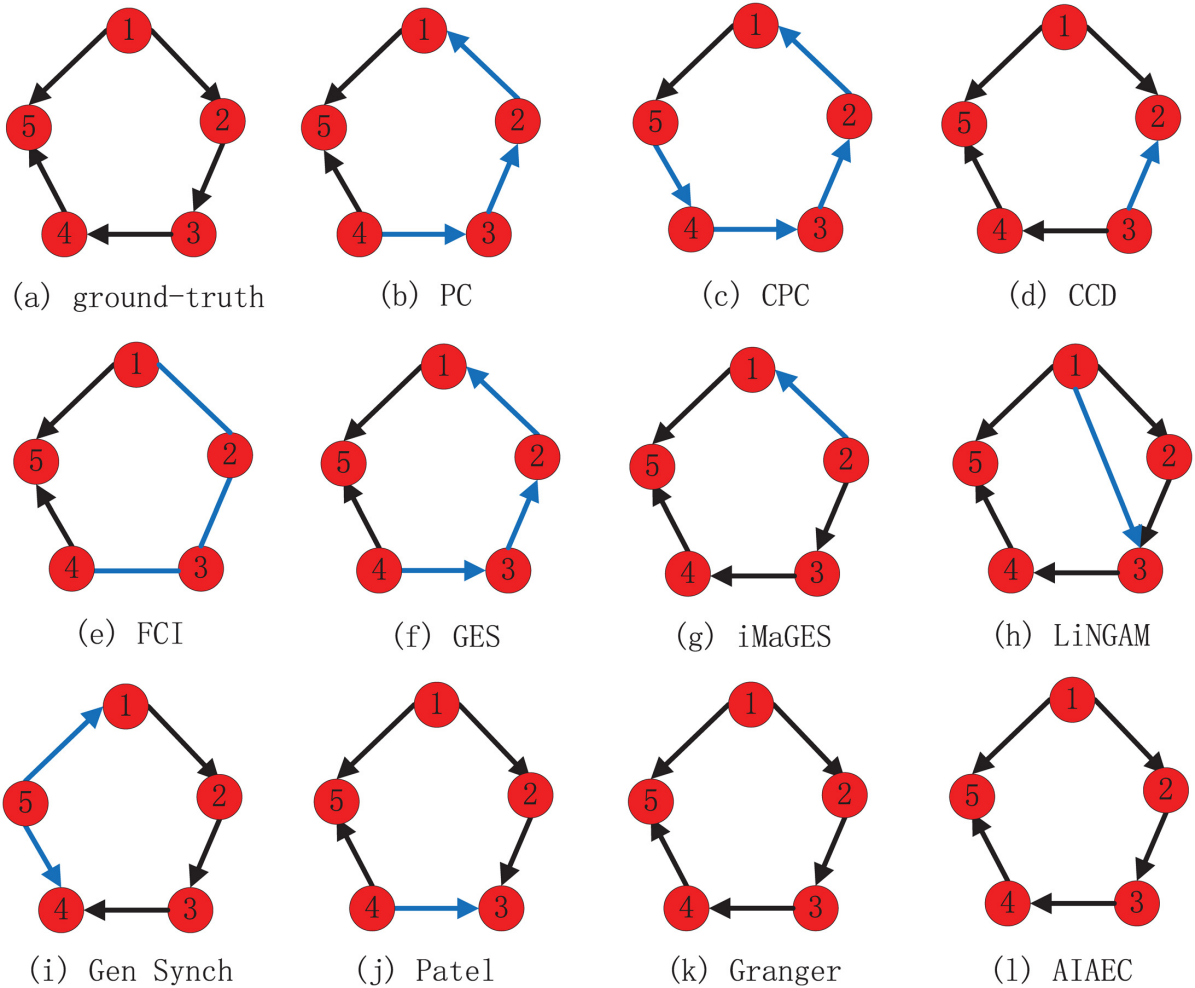
The network structures identified by various algorithms on Sim 1. (a) ground-truth. (b) PC. (c) CPC. (d) CCD. (e) FCI. (f) GES. (g) iMaGES. (h) LiNGAM. (i) Gen Synch. (j) Patel. (k) Granger. (l) AIAEC (best).

To explicitly reveal the results obtained by our algorithm, we take two networks as the examples to explain. In these two examples, two ground-truth graphs under the corresponding conditions denote the mean ground-truth networks across 50 “subjects”, other graphs show the best results detected by corresponding algorithms. Moreover, in each graph, black lines mean that the connections and directions in this graph are consistent with the ground-truth network, while the blue lines are not. Fig 6 shows the first example in Sim1, where 10 algorithms except for LiNGAM can correctly detect 5 connections, however, most of algorithms generate errors at identifying the directions. More specifically, only Granger and AIAEC algorithms shown in Fig 6(k) and 6(l) can correctly identify all directions. CCD, iMaGES and Patel algorithms can correctly identify 4 of 5 directions. As shown in Fig 6(d), 6(g) and 6(j), the error directions of CCD, iMaGES and Patel are 3 → 2, 2 → 1 and 4 → 3, respectively. LiNGAM detects an extra arc 1 → 3 shown in Fig 6(h). Gen Synch can correctly identify 3 of 5 directions, two error directions in Fig 6(i) are 5 → 1 and 5 → 4. As shown in Fig 6(b), 6(e) and 6(f), there are three error or unlabelled directions, i.e., 4 → 3, 3 → 2 and 2 → 1 for PC and GES, 4 − 3, 3 − 2 and 2 − 1 for FCI. As shown in Fig 6(c), CPC only correctly identifies a direction 1 → 5, and other directions are error.

**Fig 7:**
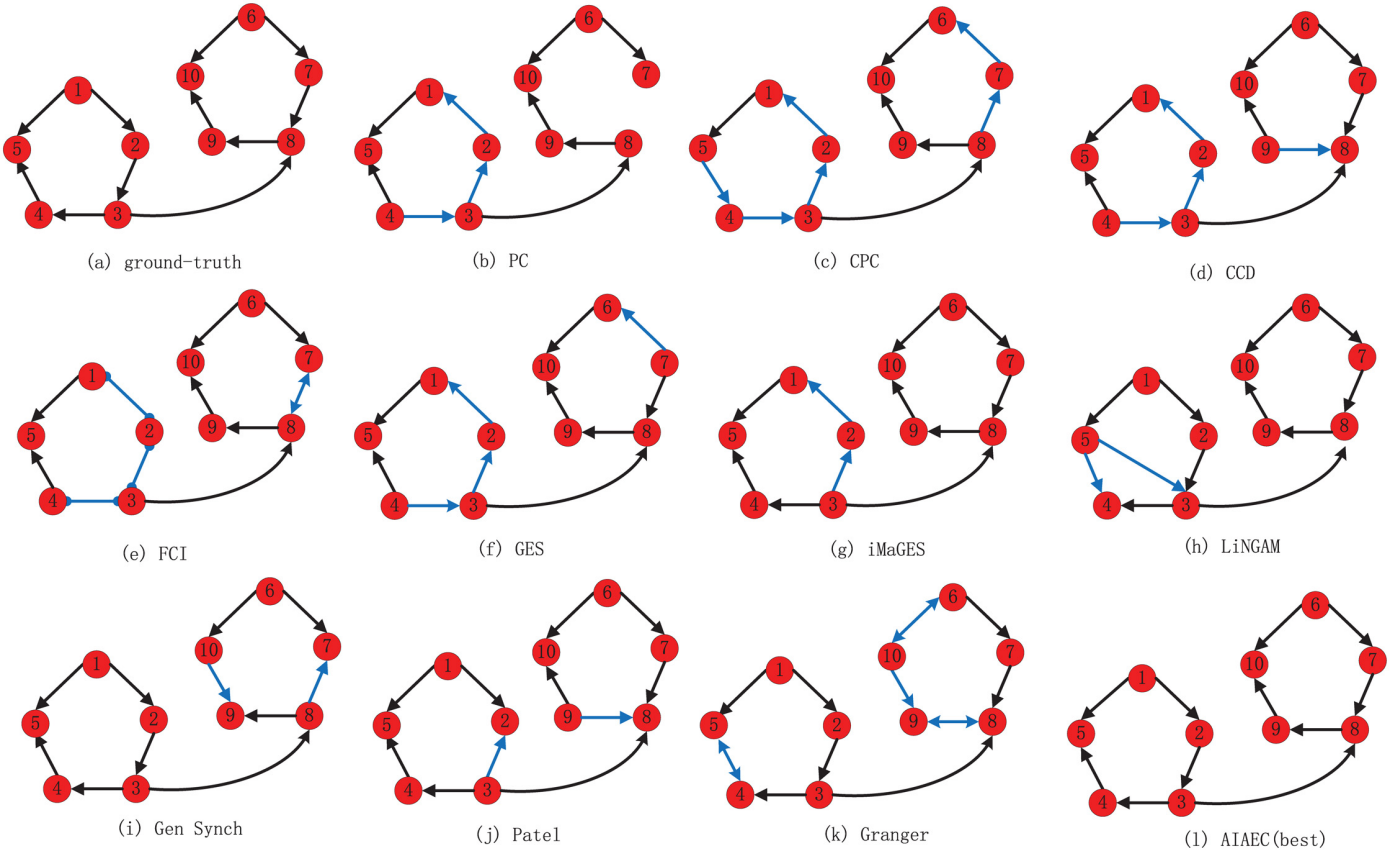
The network structures identified by various algorithms on Sim 2. (a) ground-truth. (b) PC. (c) CPC. (d) CCD. (e) FCI. (f) GES. (g) iMaGES. (h) LiNGAM. (i) Gen Synch. (j) Patel. (k) Granger. (l) AIAEC (best).

Fig 7 shows another example on Sim2, where 9 algorithms can correctly detect 11 connections except for PC and LiNGAM, in which PC loses a connection between node 7 and node 8 while LiNGAM genarates an extra connection between node 5 and node 3. For identifying the directions, AIAEC is the only algorithm which can correctly detect all directions, and other algorithms produce at least 2 mistakes. In detail, LiNGAM, iMaGES, Gen Synch and Patel generate 2 error arcs, such as 5 → 4 and 5 → 3 in Fig 7(h), 3 → 2 and 2 → 1 in Fig 7(g), 10 → 9 and 8 → 7 in Fig 7(i), and 3 → 2 and 9 → 8 in Fig 7(j). PC, CCD, FCI, GES and Granger generate 4 error arcs, they are: three error arcs (2 → 1, 3 → 2 and 4 → 3) and a losing arc 7 → 8 in Fig 7(b), four error arcs (2 → 1, 3 → 2, 4 → 3) and 9 → 8 in Fig 7(d), three undirection arcs (2 − 1, 3 − 2 and 4 − 3) and one bidirection arc 7 ↔ 8 in Fig 7(e), 4 error directions such as 4 → 3, 3 → 2, 2 → 1 and 7 → 6 in Fig 7(f), one error direction 10 → 9 and three bidirection arcs (5 ↔ 4, 9 ↔ 8, 6 ↔ 10) in Fig 7(k). Finally, as shown in Fig 7(c), CPC generates 6 error arcs: 5 ↔ 4, 4 ↔ 3, 3 ↔ 2, 2 → 1, 8 → 7 and 7 → 6.

#### Comparative whole performance

**Fig 8:**
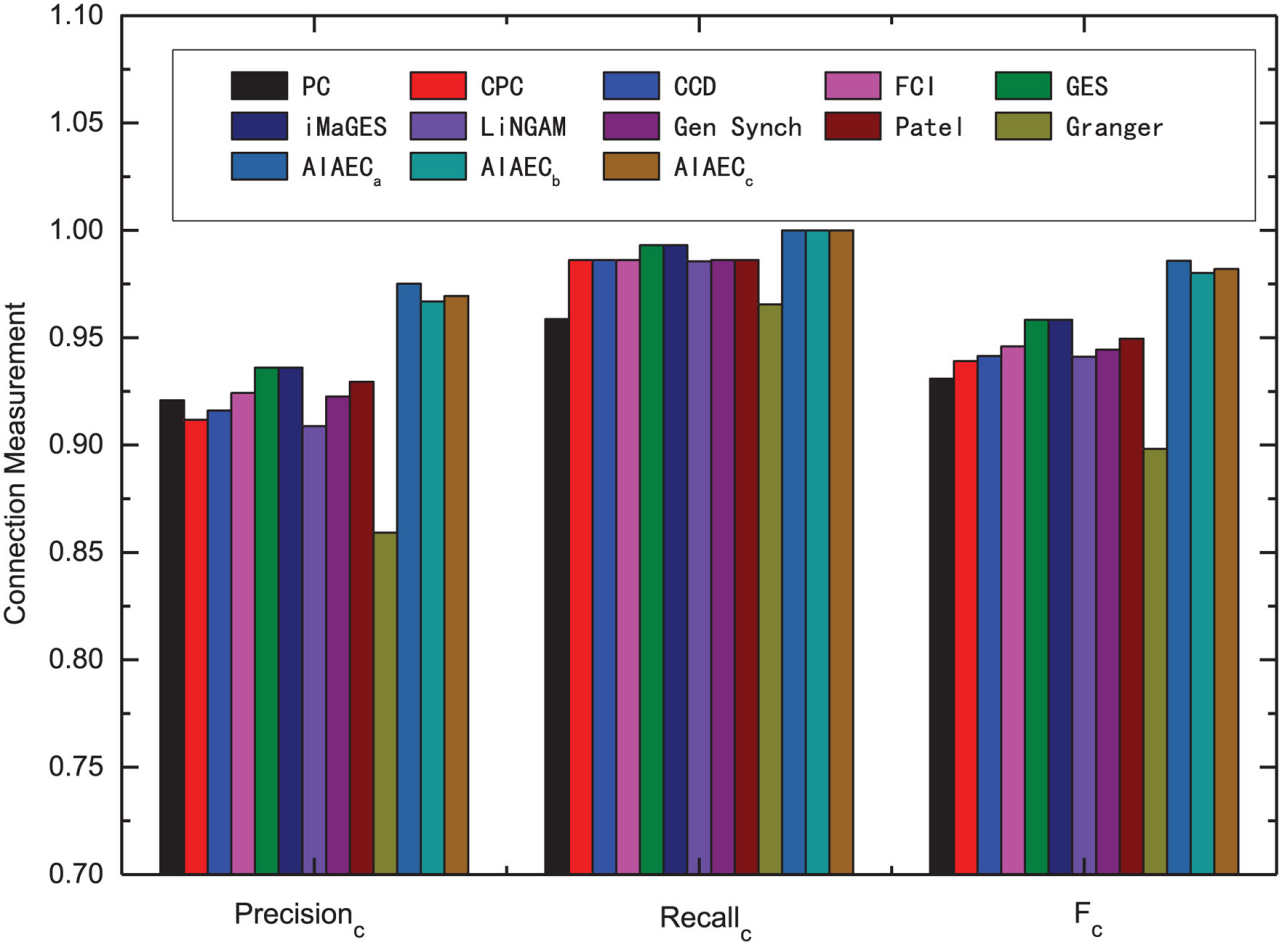
Comparative connection measurements of various algorithms over all 28 simulations.

**Fig 9:**
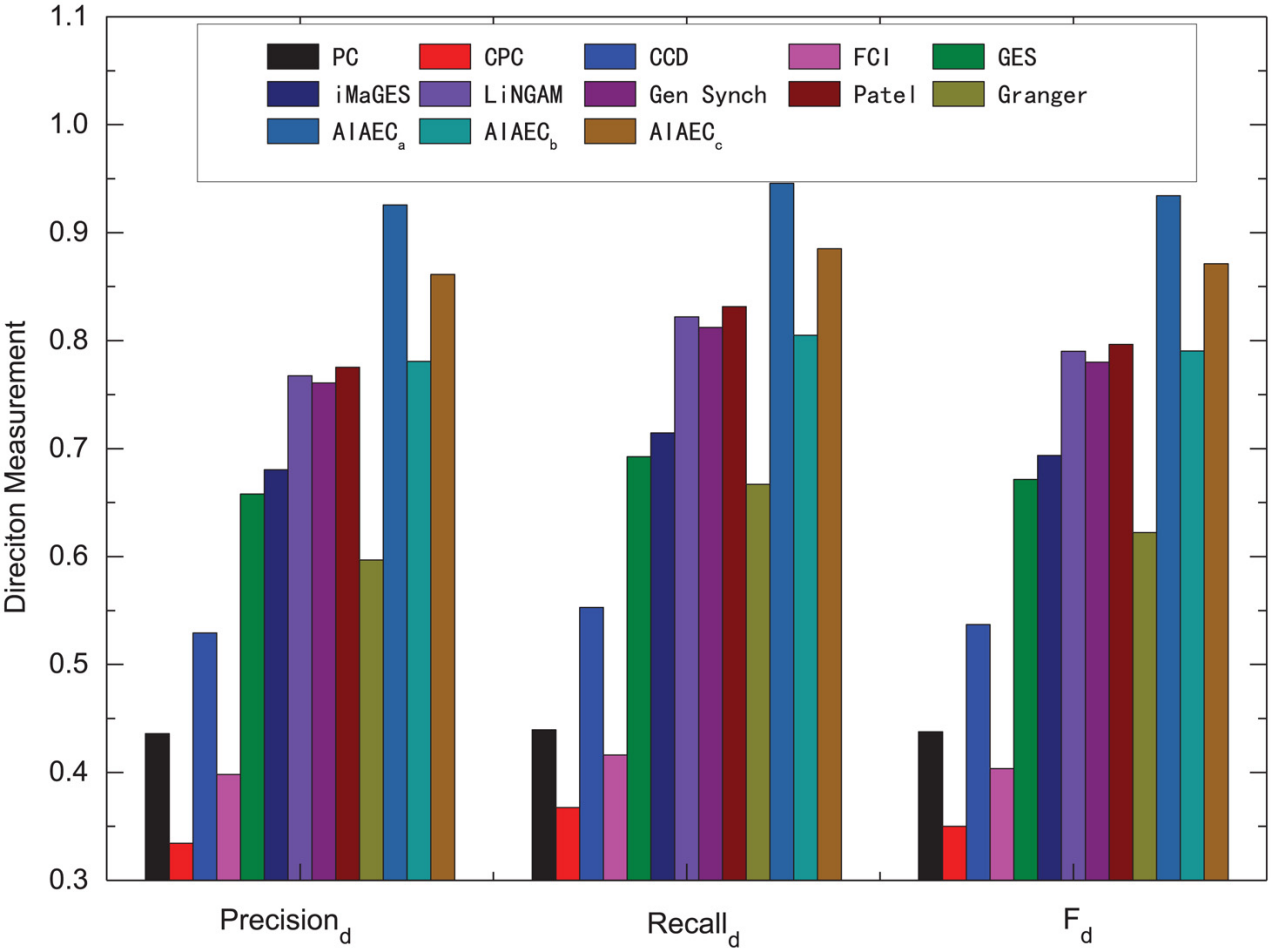
Comparative direction measurements of various algorithms over all 28 simulations.

Figs 8 and 9 show the average comparison results of these algorithms over all 28 simulations in terms of various evaluation metrics, including *Precision*_*c*_, *Recall*_*c*_, *F*_*c*_, *Precision*_*d*_, *Recall*_*d*_, *F*_*d*_ for connection and direction measurements, respectively. From Fig 8, we can conclude that AIAEC archives excellent performance on the connection measurements. In detail, it was found that AIAEC obtains the highest values of *Precision*_*c*_, *Recall*_*c*_, *F*_*c*_ in three cases though the connection measurements of all these algorithms are generally good. E.g., three *F*_*c*_ values of AIAEC are 0.9858 (*AIAEC*_*b*_), 0.9802 (*AIAEC*_*w*_), and 0.9820 (*AIAEC*_*a*_), respectively, which are 8.76%, 8.20%, and 8.38% higher than the worst value 0.8982 (Granger) in all algorithms.

Fig 9 shows the comparative direction measurements of various algorithms over all 28 simulations. We can observe that the best and mean values of AIAEC are higher than that of other algorithms while the worst value of AIAEC is only inferior to Patel. More specifically, three *F*_*d*_ values of AIAEC are 0.9342 (*AIAEC*_*b*_), 0.7905 (*AIAEC*_*w*_), 0.8711 (*AIAEC*_*a*_). *AIAEC*_*b*_ and *AIAEC*_*a*_ are 13.78%, 7.47% higher than that of the second best algorithm Patel (*F*_*d*_ = 0.7964). In other words, the performance difference on network direction is relatively large for all test algorithms where AIAEC gets the better performance in average.

## Discussion

In this paper, a new algorithm, i.e., AIAEC, is proposed, which is a global search method to learn the effective connectivity from fMRI data. In AIAEC, an effective connectivity network is mapped to an antibody, and four immune operators are then employed to perform the optimization process of antibodies, including clonal selection operator, crossover operator, mutation operator and suppression operator, and the causal connectivity network with the highest K2 score is finally output as the solution. The experimental results demonstrated that the proposed AIAEC method is superior to the other 10 algorithms in most of the 28 simulated datasets and attains the comparable performance to the best algorithms on the other cases. In the following paragraphs, the advantage and disadvantage of AIAEC will be discussed in terms of the different influential factors.

It has been demonstrated that the proposed AIAEC method is differently affected by the experimental factors. Introduction of the shared input, backward connections, stronger connections, only one stronger external input, and noise significantly decreased the performance of AIAEC, nevertheless, in these situations, AIAEC is superior to all the other 10 algorithms. Introduction of bad ROIs, cyclic connection, more connections, as well as the increasing node number also reduce the ability of AIAEC. In these cases, AIAEC still gets the comparable performance to the best algorithm (maybe Granger, Gen Synch, or Patel). When the HRF deviation is reduced, its effect on AIAEC is interacted with the TR factor. In the longer TR cases (TR = 3 s), AIAEC’s performance is reduced but still comparable to Gen Synch and superior to the other 9 methods. While in the shorter TR cases (TR = 0.25 s), AIAEC is seldom affected. When the connection strength is modulated by additional random processes, the performance of AIAEC is increased, and better than the other 10 algorithms. In addition, global mean confound has no effect on AIAEC, and even in the worst condition, AIAEC gets the best performance. The session length also has no effect on AIAEC. Following the chain of Sim26-Sim25-Sim1-Sim5-Sim7 (i.e., the session duration is increasing from 2.5 min, to 5 min, 10 min, 60 min, 250 min), it was found that AIAEC can get the stable solution of the effective connectivity structures at the relative shorter time period (i.e., 2.5 min; in this cases (Sim26), *F*_*d*_ is 0.96 averaged for AIAEC).

The current results reveal that the proposed AIAEC is better than all the other existing Bayes net methods including PC, CPC, CCD, FCI, GES, and iMaGES, and also superior or comparable to Granger, Gen Synch, and Patel. It was argued that this advantage may be mainly attributed to its strong global search ability by employing the optimization process of an antibody population. Specifically, there are three factors to enhance AIAEC’s global optimization ability. First, AIAEC is a swarm intelligent algorithm, which employs an antibody population with different initial solutions to find an optimal solution at each iteration. The swarm search mechanism can result in an extremely wide search scope, which make AIAEC to be more easily to get the higher scores. Therefore, AIAEC is better than the other two score-based Bayes net algorithms (GES and iMaGES) when there is no added factor in Situation 1. Second, AIAEC utilizes a random search mechanism with some immune operators to ensure the diversity of solutions. For instance, crossover and mutation operators of antibodies enhance the AIAEC’s global search capability and avoid trapping into local optimum. This characteristic makes AIAEC perform well in Situation 2, especially in Sim25 and Sim26 where the sessions are short. As we all know, when the session is short, the information of subjects will be less, which may bring more difficulty, for the search algorithm to find a good solution. By these immune operators, AIAEC can maintain the diversity of solutions, which help AIAEC find the best solution in the situation with less data. Third, AIAEC adopts a memory set in generating an initial population. The memory mechanism can keep the useful information of excellent antibodies in the ancestors and guide the evolution of descendants in stochastic evolutionary process, which reduces the repeated and blind exploring in a random searching and accelerates the convergence speed of AIAEC. For instance, in Situation 10, AIAEC can correctly detect all network connections and entirely identify all connection directions in which the memory mechanism plays an important role in using the history information and avoiding solution degeneration.

The noise-tolerance ability of AIAEC may also contribute to its performance. When various noises (not limited to the signal noise level) are added into data, many algorithms are seriously affected, but AIAEC still maintains its performance. This may be attributed to the fact that AIAEC simulates an immune mechanism which not only takes into account local connection between two nodes, but also judges the global impact of each connection on the whole network during the learning process of network structures. In other words, just like iMaGES, AIAEC searches in the space of the overall graph over the ROIs, but not in the space of what the weights or strengths of these causal relationships are for each connection across subjects [20]. The identifying capabilities of AIAEC and iMaGES are confirmed in Situation 8 and Situation 12, where the performance of many algorithms were worse while AIAEC and iMaGES still performed well. The good noise tolerance of AIAEC was also reflected in Situation 14, where AIAEC always keeps a good identifying ability whether the noises are added.

Another advantage of AIAEC is its self-adaptability. Many algorithms are highly dependent on their parameter setting and threshold selection [8]. That is, the improper threshold or parameter value may induce the bad results. Thus, many algorithms usually require a considerable amount experiments to determine the value of parameters and thresholds on different datasets. To objectively demonstrate the solving abilities of 10 comparison algorithms, we have tried to make them have the best parameter values in our experiments. As shown in Tables 2 to 8, we keep changing the parameters of some algorithms to adapt to different simulations. Though AIAEC also has some parameters, its performance is not sensitive to the parameter values. This is because that AIAEC simulates a kind of heuristic search mechanism, artificial immune, to search the network connection structure with the best score. The artificial immune mechanism has self-learning and adaptive abilities, which can not only keep the balance between exploitation and exploration processes, but also realize the perfect combination of global searching and local searching in the available solution space. Therefore, AIAEC does not need to manually set a threshold to determine whether there is a connection between two nodes, and it is able to automatically learn a network structure without manual interventions from various datasets.

There are still some limitations for AIAEC. First, AIAEC requires discretized data as inputs, so it needs an extra data preprocessing step on every simulated dataset. How to overcome the above limitation will be important to expand the application of AIAEC. Second, AIAEC cannot guarantee the performance of identifying directions when there is a cyclic causality in a brain network (e.g., in Sim14). The reason is that the cyclic causality breaks the acyclic assumptions for many Bayes net methods including AIAEC, which also has been indicated by Smith et al. (2011).

## Conclusion

This paper presents a new method for learning effective connectivity network structure from fMRI data, i.e., AIAEC. The effectiveness of AIAEC has been experimentally verified. Moreover, AIAEC is superior to the other existing 10 algorithms in the majority of the datasets. The advantages of AIAEC (e.g., shorter session duration and higher noise-tolerance ability) imply that it is promising for practical applications in the neuroimaging studies of pediatric, geriatric subjects and neurological patients.
